# The Extension Arm Design Method Based on a Two-Bar Tension Stretchable Mechanism

**DOI:** 10.1155/abb/3313533

**Published:** 2025-02-22

**Authors:** Song Gao, GuoFu Zhang, Yue Zhao, XiaoHui Yu, JianWei Sun

**Affiliations:** ^1^School of Mechatronic Engineering, Changchun University of Technology, Changchun 130022, China; ^2^College of Mechanical and Automotive Engineering, Humanities and Information Changchun University of Technology, Changchun 130400, China

**Keywords:** deployable mast, stability analysis, two-bar tensegrity structure, unit extension displacement test

## Abstract

The deployable and foldable structure is a unique structural system that provides convenience in terms of transportation and expansion. Although the current deployable mast can transition between closed and extended structures, it lacks stability during movement and does not have the ability to recover on its own after contracting. Hence, improving the stability of deployable mechanisms while enabling them to self-restore has become an essential area for development. Spatial deployable mast expansion and contraction properties are essential for aerospace. In this paper, based on a two-bar and four-cable tensegrity structure, the research was conducted. A new spatially deployable mast structure with zero Poisson's ratio and self-stability, as well as self-adaptability, is proposed. In comparison to existing space deployable masts, the new deployable masts incorporate a tensegrity structure feature that enables them to recover autonomously to their initial state in a contracted state. Firstly, an innovative design based on a two-bar, four-cord tensegrity structure is conducted, which evolves into a two-bar and four-cable tensegrity structure with zero Poisson's ratio by increasing the longitudinal axis to restrict the structure's degrees of freedom and combines with the force density method to analyze the stability of the structure and derive the optimal dimensional parameters. The positive definiteness of the stiffness matrix *Q* (*K*) demonstrates the exceptional stability of the mechanism. Secondly, the load-bearing characteristics of the mechanism were verified utilizing ABAQUS (DS, France) software. Besides, the minimum compressive stiffness of the new space deployable mast for locking three rails compared to locking one rail is 12 × 10^8^ N/m, as derived from ABAQUS (DS, France) analysis. Ultimately, a prototype was developed through the application of three-dimensional (3D) printing technology. The force–strain experiment was conducted using both a pressure testing machine and a universal testing machine, and the resulting images demonstrated the findings. The experimental results demonstrate that the novel deployable mast structure has self-recovering and self-stabilizing capabilities. This research pushes the frontiers of deployable mast multifunctionality as well as significantly expands the application domain of tensegrity structures to the aerospace sector.

## 1. Introduction

The increasing urgency for sizable spacecraft is driven by the swift advancements in space technologies, encompassing deep space exploration, Earth observation, ocean exploration, and military reconnaissance. Due to the limitations of the launch vehicle in terms of load volume, the fulfillment of the mission requirements is unattainable for large-scale nonfolding spreading structural bodies. Moreover, the deployable mast requires little workspace in the stowed state and is capable of being deployed while stowed to the deployed state during operation, which circumvents the storage space constraint for launch vehicles and facilitates transportation, consequently making the realization of large-scale spacecraft with space-deployable mechanisms possible [1–3]. The deployable mast has the advantages of lightweight, small volume, strong load-bearing capacity, flexible expansion, etc., and it is a kind of complex space structure [4]. Furthermore, the deployable mast can be classified into broad categories following its structural support and deployment: thin-walled tubular space deployable mast, telescopic deployable mast, compliable deployable mast, and folding articulated square deployable mast. Among them, Becchi and Dell'Amico [5] have designed and developed a deployable mast mechanism with a stowed height of up to 2.4 m, consisting of seven socketed basic units, which have been successfully applied to tethered satellites. Additionally, the able deployable articulated mast, developed by AEC-Able [6], has a diameter of 1.12 m and a length of up to 60 m in the deployed state. Except for that, Arita et al. [7] design a spatially expandable quadrilateral mechanism based on the principle of folding and deformation. Meanwhile, Choi et al. [8] give a schematic design of a high-precision quadrangular support arm applied to space optical telescopes. Subsequent developments in space deployable masts must prioritize the benefits of enhanced stability, cost-effectiveness, lightweight construction, and improved performance. The spreadable mechanism with tensegrity structure has the characteristics of lightweight, high stability, and readily spreadable.

A tensegrity structure is a self-equilibrating, prestressed articulated structure composed of individual rod members experiencing only compression forces and continuous cable members undergoing only tension forces. This simple construction principle gives the tensegrity structure new mechanical properties, including lightweight and high material availability owing to uniaxial loading [9–12]. Besides, the tensegrity structure structure is included in the category of space structures with small strains and large displacements. Moreover, the tensegrity structure takes lightweight and flexible cable members as the main body, augmented by regional rigid rod constituents, which only require the application of appropriate prestressing in the members to realize the rigidization of the structure, without external support and load, with adjustable stiffness, controllable shape, readily foldable and spreadable, along with other notable [13–15]. Zhang, Zhao, and Feng [16–18] used prismatic tensegrity structure and polyhedral tensegrity structure as modules to construct a large-scale structure. Liu et al. [15] proposed a tensegrity structure framework, and the tensegrity structure structural assemblies of deployable structures are likewise adopted as masts [19, 20], bridges [21–23], platforms [24–27], and antennas [28–30]. Yildiz and Lesieutre [31] employed a particle swarm optimization algorithm to design a lightweight and high stiffness class 2 triple rod tensegrity structure deployable mast. Furthermore, Kan et al. [32] obtained a tensegrity structure deployable mast with 10 tetragonal prismatic units axially connected by building a multinodal collector cable, which requires less effort to implement than the traditional cable rod mechanism. In addition, Zhang et al. [33]constructed a variety of one-dimensional tensegrity structure structures with deformable tensegrity structure units with end plates, expanding the applications of the deployable mast. Except for that, Tibert [6] proposed a design for an arm-like tensegrity structure in which the rod members exhibit elastic bending properties. The theory underlying this design is to preserve the bowed condition of the bar member utilizing a tensioning cable and to return the bar member to an upright state by releasing the tensioning cable in response to an elastic force. By means of an internally supported cable network structure, this design concept implements the principles of tensegrity structures to create the desired arm-like tensegrity structure. Veuve, Sychterz, and Smith [34, 35] investigated a foldable spreading method for second-order armchair tensegrity structure structures. The rod members of this spreadable structure are entirely rigid and not foldable, while some of the cable members are connected in series to drive the mechanism for folding, and certain cable segments are substituted with springs. Due to the complexity of the relative motion between the connected rod members, ball joints or universal joints are employed for the connection of the rod members. Averseng and Dubé [36] spliced multiple four-bar tensegrity structure units laterally to form an arm-like tensegrity structure structure. By utilizing the diagonal cable of the tensegrity structure unit as a driving cable, by shortening the driving cable, a predetermined state is induced in the folded arm structure. Fadeyev et al. [37] built a two-stage tensegrity structure robotic arm experimental platform, which is encompassed by eight OptiTrack cameras to capture the displacement information of the nodes through optical motion. Additionally, the experimental platform facilitates strong validation of the experimental data as well as theoretical analysis of the tensegrity structure robotic arm prototype, and the experimental platform can be utilized to collect data for additional tensegrity structure research. In addition to the innovation of mechanism functions in the aerospace industry, there are also stringent requirements for material selection [38–41]. Hallad et al. [42] made a significant contribution to material selection for the aerospace industry by incorporating lead oxide into traditional concrete, thereby creating a novel composite material. The compressive and deflection properties of composite materials containing lead oxide were evaluated using six different mixed specimens. Furthermore, the feasibility of this material was assessed through physical experiments and ABAQUS (DS, France) simulations, ultimately confirming its viability [43]. Additionally, numerous bionic model databases have been established, providing an essential theoretical foundation for the development of mechanical properties [44]. These studies exhibit inherent challenges commonly associated with conventional spatially deployable mechanisms, such as excessive degrees of freedom, internal stress interference during motion, limited stability, and a lack of inherent self-recovery. To tackle this critical issue, a new type of deployable mast structure in this paper based on the two-bar tensioning integral structure was innovatively constructed according to the traditional triangular prism deployable mast. By integrating the benefits of a lightweight tensegrity structure that is a self-stabilizing, self-recovering, and self-stabilizing framework with a deployable mast framework in an organic manner, the design model of the space-deployable deployable mast of tensegrity structure is established. Besides, the excellent performance of the structure is verified by in-depth analysis of stability, ADAMS (MSC, USA) dynamics, ABQUS finite elements, and unit stretch displacement tests as well as verification by a universal testing machine.

### 1.1. Mapping of Tensioned Monolithic Structural Units

**Figure 1:**
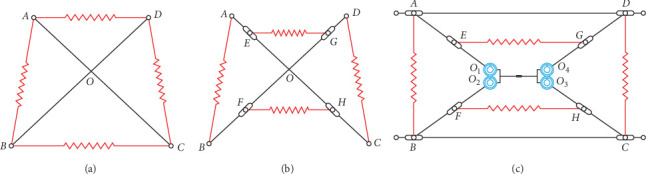
Sketch of the structure of the tensionable unit: (a) a two-bar, four-cable tensioning system; (b) an improved tension integral design; and (c) an innovative overall tensioning mechanism.

The excellent reconfigurability and self-stability of the tensioned structure exacerbate the requirement for a reduced mass and more manageable deformation, and accordingly, a new type of deployable mast is constructed based on the tensioned structure. To improve the stability and self-recovery characteristics of the tensioned structure, we incorporate the fixed section characteristics of the spreadable mechanism into the configuration design of the tensioned structure, which involves the integration of the tensegrity structure with the weaknesses of the spreadable mechanism's lack of flexibility and self-recovery capability to compensate. As depicted in Figure 1, the configurations include: (a) a two-bar, four-cable tensioning system; (b) an improved tension integral design; and (c) an innovative overall tensioning mechanism. Figure 1a indicates the two-bar tensioning model, and the design of the deployable mast of the spreadable mechanism based on the two-bar tensioning structure proposed in this paper is an optimized mechanism for the deployable mast of the spreadable mechanism by altering the relative positions of the tension member (tension spring) and the compression member (compression rod) in the two-bar and four-cable tensegrity structure. Moreover, to improve the stability and at the same time easier to assemble, it is imperative to guarantee that the lengths of *AD* and *BC* shown in Figure 1a remain unchanged during the movement of the mechanism, and the specific optimization process is as follows: as illustrated in Figure 1a, we displace the upper two sides of the tension spring endpoints *A a*nd *D* downward and the lower two sides of the tension spring endpoints *C* and *D* upward within the plane of the two-bar and four-cable tensegrity structure by a specific distance to achieve Figure 1b. The new deployable mast unit structure revealed in Figure 1b consists of upper-side tension spring endpoints *E* and *G* and lower-side tension spring endpoints *F* and *H*. Even though the structure demonstrated in Figure 1b can ensure the balance of forces at *EG* and *FH* during the extension process and maintain the steady state of the overall tensioning structure, the trajectories of points *A* and *D* deviate from the design of the constant section. To solve the problem of insufficient stiffness of the tensegrity structure itself, we use mechanical members to cooperate to enhance the overall structural stiffness, and the specific operation is as follows: with the intersection point *O* of *AC* and *BD* as the central point, divide the rod *AC* and *BD* into four rods *AO*_1_, *BO*_2_, *CO*_3_, *DO*_4_, with a group containing two gear sets with gears of the identical transmission ratio to match *AO*_1_ and *BO*_2_ with each other, and similarly *CO*_*3*_ and *DO*_4_ are constrained, and subsequently, the gear sets *O*_1_*O*_2_ and *O*_3_*O*_4_ are interrelated by linear guides to acquire the structure demonstrated in Figure 1c. To meet the theoretical requirements of fixed section design, where *AD* and *BC* are fixed length rods, the role of which is to allow points *A* and *D* to move in a linear trajectory parallel to the *y*-axis direction, in the state of stacking multiple groups of deployable mast units to guarantee in their entirety the stability properties of the mechanism that the fixed section design imparts; during the movement of the mechanism, tension springs *EG, FH*, *AB*, and *CD* are self-stabilized by the force, in line with the theoretical requirements of two-bar and four-cable tensegrity structure. Furthermore, the theoretical requirements of the mechanism are met, and the gears and slides in the mechanism ensure the synchronization of the extension arms during extension and contraction, avoiding the movement errors induced by single-sided asynchrony. Certain deformation variables characterize the resting state of the tensioning springs *EG*, *FH*, *AB*, and *DC*.

## 2. Methodology

**Figure 2:**
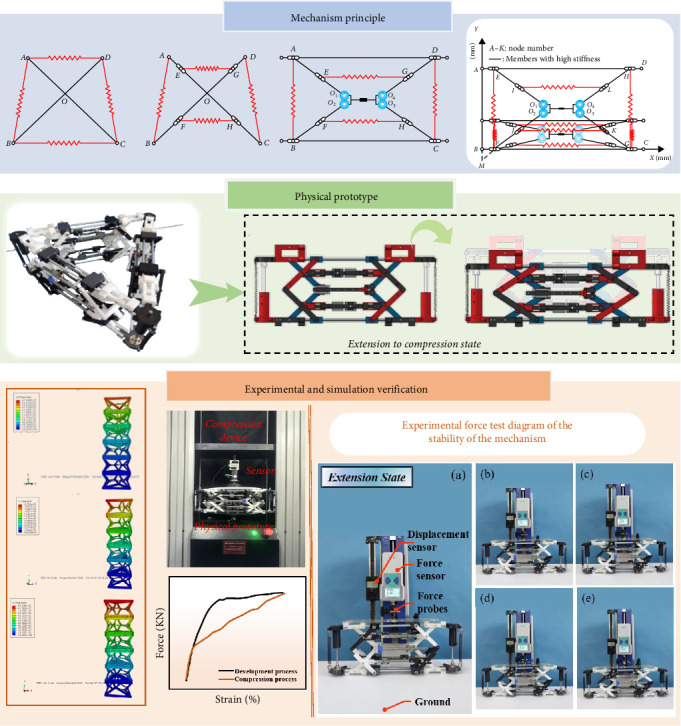
Flowchart of the work process.

The entire research process is structured within the framework of a methodology, as depicted in Figure 2.

### 2.1. Stability Analysis of Tensioned Structures

Stability analysis was conducted based on the equivalent model of the tensioned structure obtained in the previous section. Besides, the *O*–*xy* coordinate system was established, and the parameters were determined in accordance with the proportions of the studied conventional deployable mast structure dimensions as illustrated in Table 1.

**Figure 3:**
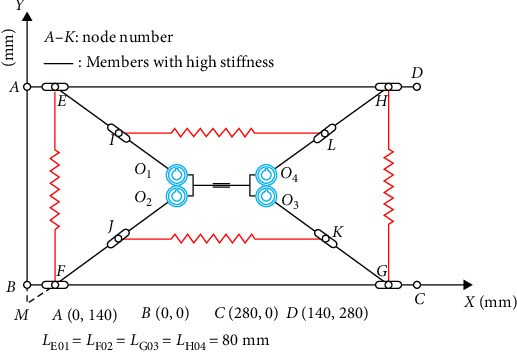
*O*–*xy* coordinate diagram of the deployable mast structure.

The initial state of the selected tensioned integral structural equivalent model is introduced into the coordinate system as shown in Figure 3. The positions of points *E*, *G*, *F*, and *H* change with the movement of the structure, while *AB* and *CD* represent the initial length, each unit maintaining its original length.

The stability [44] of the tensegrity structure is strikingly similar to the tangential stiffness matrix *K*. If the tangential stiffness matrix *K* is positive, subsequently, the mechanism is stable and vice versa. Consequently, the stability of the overall two-bar tensioned and spreadable mechanism model is assessed by the positivity of the tangential stiffness matrix *K*.

The tangential stiffness matrix *K* indicates the sum of the elastic stiffness matrix *K^E^* and the geometric stiffness matrix *K^G^*:

(1) $$\begin{array}{c} K=K^{E}+K^{G}. \end{array}$$

To determine whether *K* is positive or not, it is imperative to ascertain whether *K^E^* and *K^G^* are positive or not, respectively. Moreover, the two-bar and four-cable mechanism pertains to the class 2 structure of R. Connelly and W. Whiteley, and class 2 belongs to the category of “super stability”; therefore, in accordance with the study of R. Connelly, it is feasible to ascertain directly, whether *K^G^* is semi-positive, that is, for any two-bar. The nonzero displacement mode of a four-cable tensionable mechanism *d* is shown as follows:

(2) $$\begin{array}{c} K^{E}=\left(AL^{\left(-1\right)}\right)G{\left(AL\right)}^{T}. \end{array}$$

*A* indicates the equilibrium matrix of the two-bar tensionable spreading mechanism, *L* represents the diagonal matrix of the initial lengths of the two-bar tensionable spreading mechanism units, and *G* demonstrates the diagonal matrix of the deformation of the stiffness of the two-bar tensionable overall structural units. The three components can be analyzed and calculated separately to determine the positivity of the elbow tensioning overall structural elastic stiffness matrix *K^E^*:

(3) $$\begin{array}{c} \begin{array}{cc} L & =\mathrm{diag}\left(\begin{array}{cccc} l_{AB} & l_{AD} & l_{CD} & \begin{array}{cc} \begin{array}{cc} l_{BC} & l_{AC} \end{array} & l_{BD} \end{array} \end{array}\right)\\ \; & =\mathrm{diag}\left(\begin{array}{cccc} 140 & 280 & 140 & \begin{array}{cc} \begin{array}{cc} 280 & 260 \end{array} & 260 \end{array} \end{array}\right). \end{array} \end{array}$$

**Figure 4:**
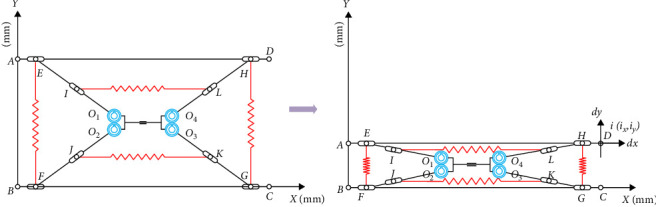
Length variation diagram.

Define *x*, *z* (*∈ R*^4 × 4^) to represent node *i* and the vector of node coordinates in the *x* and *z* directions, the external force to which the rod unit or spring unit *ij* (*ij* = 1, 2, 3, 4, 5, 6,) is subjected, the change in length of the rod unit or spring unit *ij* under the external force, and the amount of change in length of the rod unit or spring unit is *l*_*ij*_, as well as the change in length which is indicated in Figure 4.

Define *q* as the ratio of the force in that rod unit or spring unit *ij* to the amount of change in the rod unit or spring unit *l*_*ij*_; subsequently, *q* = (*q*^*1*^, *q^2^, q^3^, q^4^, q^5^, q^6^*) is the matrix of force density coefficients for the tensionable spreading mechanism. Consequently, the force density matrix *Q∈R*^*6*^of a tensioned and extendable structure can be acquired, and then *Q* is as follows:

(4) $$\begin{array}{c} Q=\mathrm{diag}\left(q\right). \end{array}$$

This results in a system of equilibrium equations for all nodes of the tensionable mechanism in each direction, as illustrated as follows:

(5) $$\begin{array}{c} C^{T}QC_{x}=P_{X}, \end{array}$$

(6) $$\begin{array}{c} C^{T}QC_{z}=P_{Z}. \end{array}$$

In the equation, *P*_*x*_, *P*_*z*_ (*∈R*^4 × 4^) represents the external force vectors acting in the direction of nodes *i* and *x* and *z*, respectively.

To simplify the equation, the matrix *E∈R*^4 × 4^ is defined as follows:

(7) $$\begin{array}{c} E=C^{T}QC. \end{array}$$

Taking into account the characteristics of the deployable mast unit structure, the shape of the deployable mast can be determined by the coordinates of the corresponding union points *i* and *j*. Hence, Equations (5) and (6) may be expressed as follows:

(8) $$\begin{array}{c} E_{x}=P_{x}, \end{array}$$

(9) $$\begin{array}{c} E_{x}=P_{x}. \end{array}$$

Combine Equations (8) and (9) as follows:

(10) $$\begin{array}{c} E\left[\begin{array}{cc} x & z \end{array}\right]=C^{T}QC\left[\begin{array}{cc} x & z \end{array}\right]=\left[\begin{array}{cc} P_{x} & P_{z} \end{array}\right]. \end{array}$$

In the equation, [*x z*] denotes the coordinate matrix of the deployable mast structure point *i*. Bringing Equation (4) into Equation (10), combining the structure of deployable mast cells yields the subsequent nodal equilibrium equation:

(11) $$\begin{array}{c} Aq=\left(\begin{array}{c} C^{T}\mathrm{diag}\left(C_{x}\right)\\ C^{T}\mathrm{diag}\left(C_{z}\right) \end{array}\right)q=\left[\begin{array}{cc} P_{x} & P_{z} \end{array}\right]. \end{array}$$

Define *A∈R*^8 × 8^ in the equation as the balanced matrix of the deployable mast mechanism.

According to the schematic diagram of the mechanism of the tensionable spreading mechanism, as indicated in Figure 2. Four spring units and two intersecting rod units comprise the model's six-unit bodies and four nodes in total. The four nodes are *A*, B, *C*, and *D*, corresponding to 1, 2, 3, 4, 5, and 6 unit bodies in that order, respectively, *AB*, *BC* (*FH*), *CD*, *AD* (*EG*), *AC*, and *BD*.

According to the definition of the association matrix *C*, the table of the nodal association matrix of the two-rod tensor as a whole is obtained, as demonstrated in Table 2.

The following equation represents the correlation matrix of the two-bar tensioned integral expandable mechanism:

(12) $$\begin{array}{c} C=\left[\begin{array}{cccc} 1 & -1 & 0 & 0\\ 1 & 0 & 0 & -1\\ 0 & 0 & 1 & -1\\ 0 & 1 & -1 & 0\\ 1 & 0 & -1 & 0\\ 0 & 1 & 0 & -1 \end{array}\right]. \end{array}$$

Based on the structural characteristics of the two-bar tensioning monolithic deployable mechanism, the dimensional characteristic parameters of the mechanism were ascertained. *BC* = 280 mm; *AB* = 140 mm; on the basis of the deployable size parameters of the two-bar tensioning monolithic, the state element length was *l*_*ij*_ (*i* = 1, 2, 3, *j* = 1, 2, 3, 4, *i* <*j*); and each element acted by external forces corresponds to *q*. The two-bar tensioning monolithic deployable mechanism comprises six components. Subsequently, the force density coefficient matrix of the two-bar tensioned integral deployable mechanism is *q* = {*q_1_, q_2_,…q_6_*}^T^.

Consequently, the force density matrix *Q∈R*^6 × 6^ for the entire two-bar tensioned integral expandable mechanism may be expressed in a subsequent manner: *Q* = diag (*q*) = diag (*q*^1^, *q*^2^, *q*^3^, *q*^4^, *q*^5^, *q*^6^,) and thus, the matrix *E* = R^4 × 4^ is acquired as follows:

(13) $$\begin{array}{c} \begin{array}{cc} E & =C^{T}QC\\ \; & =\left(\begin{array}{cccccc} 1 & 1 & 0 & 0 & 1 & 0\\ -1 & 0 & 0 & 1 & 0 & 1\\ 0 & 1 & 1 & -1 & -1 & 0\\ 0 & -1 & 0 & 0 & 0 & 1 \end{array}\right)\left(\begin{array}{ccccc} q_{1} & \; & \; & \; & \;\\ \; & q_{2} & \; & \; & \;\\ \; & \; & q_{3} & \; & \;\\ \; & \; & \; & q_{4} & \;\\ \; & \; & \; & \; & q_{5} \end{array}\right)\left(\begin{array}{cccc} 1 & -1 & 0 & 0\\ 1 & 0 & 0 & -1\\ 0 & 0 & 1 & -1\\ 0 & 1 & -1 & 0\\ 1 & 0 & -1 & 0\\ 0 & 1 & 0 & -1 \end{array}\right) \end{array}, \end{array}$$

(14) $$\begin{array}{c} E=\left(\begin{array}{cccc} q_{1}+q_{2}+q_{5} & -q_{1} & -q_{5} & -q_{2}\\ -q_{1} & q_{1}+q_{4}+q_{6} & -q_{4} & -q_{6}\\ -q_{5} & -q_{4} & q_{3}+q_{4}+q_{5} & -q_{3}\\ -q_{2} & -q_{6} & -q_{3} & q_{2}+q_{3}+q_{6} \end{array}\right). \end{array}$$

By focusing solely on the properties of the tensioning mechanism and disregarding the influence of external forces and self-weight, the following can be deduced using Equation (8):

(15) $$\begin{array}{c} E\left[\begin{array}{cc} x & y \end{array}\right]=\left[\begin{array}{cc} 0 & 0 \end{array}\right]. \end{array}$$

By substituting Equation (12) into Equation (13), the following formula can be obtained:

(16) $$\begin{array}{c} \left(\begin{array}{cccc} q_{1}+q_{2}+q_{5} & -q_{1} & -q_{5} & -q_{2}\\ -q_{1} & q_{1}+q_{4}+q_{6} & -q_{4} & -q_{6}\\ -q_{5} & -q_{4} & q_{3}+q_{4}+q_{5} & -q_{3}\\ -q_{2} & -q_{6} & -q_{3} & q_{2}+q_{3}+q_{6} \end{array}\right)\left(\begin{array}{cc} 1 & 140\\ 280 & 140\\ 0 & 0\\ 280 & 0 \end{array}\right)=\left(\begin{array}{cc} 0 & 0\\ 0 & 0\\ 0 & 0\\ 0 & 0 \end{array}\right), \end{array}$$

Due to *q*_1_ = *q*_3_ = *q*_4_ = *q*_6_ = 1, *q*_2_ = *q*_3_ = −1*Q* = diag (*q*) = diag (1 − 11 − 111), and force density matrix, which is got by judgment *q_1_, q_3_, q_4_, q_6_ >0, q_2_, q_3_ <0*, and the geometric model and structural parameters of the two-bar tensioned integral extendable mechanism exhibit a satisfactory relationship.

*G* indicates the diagonal matrix of stiffness deformation of the two-bar tensioning deployable mechanism element, the stiffness deformation of the spring element of the two-bar tensioning deployable mechanism represents the force density of the spring element, and the rod element of the two-bar tensioning deployable mechanism illustrates the rigid structure. Consequently, stiffness deformation of the rod element of the two-bar tensioning deployable mechanism is ignored as, thus, it can be obtained:

(17) $$\begin{array}{c} G=\mathrm{diag}\;\left(\begin{array}{cccccc} q_{1} & q_{2} & q_{3} & q_{4} & 0 & 0 \end{array}\right), \end{array}$$

(18) $$\begin{array}{c} AL^{-1}=\left(\begin{array}{cccccc} -280 & -280 & 0 & 0 & 0 & 0\\ 280 & 0 & 0 & 280 & 0 & 560\\ 0 & 0 & -280 & -280 & 0 & 0\\ 0 & 280 & 280 & 0 & 0 & 560\\ 0 & 140 & 0 & 0 & 140 & 0\\ 0 & 0 & 0 & 140 & 0 & 140\\ 0 & 0 & 0 & -140 & -140 & 0\\ 0 & -140 & 0 & 0 & 0 & 140 \end{array}\right){\left(\begin{array}{cccccc} 140 & \; & \; & \; & \; & \;\\ \; & 280 & \; & \; & \; & \;\\ \; & \; & 140 & \; & \; & \;\\ \; & \; & \; & 280 & \; & \;\\ \; & \; & \; & \; & 260 & \;\\ \; & \; & \; & \; & \; & 260 \end{array}\right)}^{-1}, \end{array}$$

(19) $$\begin{array}{c} G=\left(\begin{array}{cccccc} 1 & \; & \; & \; & \; & \;\\ \; & 1 & \; & \; & \; & \;\\ \; & \; & 1 & \; & \; & \;\\ \; & \; & \; & 1 & \; & \;\\ \; & \; & \; & \; & 0 & \;\\ \; & \; & \; & \; & \; & 0 \end{array}\right), \end{array}$$

(20) $$\begin{array}{c} \begin{array}{cc} K^{E} & =\left(AL^{-1}\right)G{\left(AL^{-1}\right)}^{T}\\ \; & =\left(\begin{array}{cccccccc} 5000 & -40000 & 0 & -10000 & -0.5 & 0 & 0 & 0.5\\ -40000 & 50000 & -10000 & 0 & 0 & 0.5 & -0.5 & 0\\ 0 & -10000 & 50000 & -4000 & 0 & -0.5 & 0.5 & 0\\ -10000 & 0 & -40000 & 50000 & 0.5 & 0 & 0 & -0.5\\ -0.5 & 0 & 0 & 0.5 & 0.25 & 0 & 0 & -0.25\\ 0 & 0.5 & -0.5 & 0 & 0 & 0.25 & -0.25 & 0\\ 0 & -0.5 & 0.5 & 0 & 0 & -0.25 & 0.25 & 0\\ 0.5 & 0 & 0 & -0.5 & -0.25 & 0 & 0 & 2.5 \end{array}\right) \end{array}. \end{array}$$

The eigenvalues of *K*^*E*^ are obtained by MATLAB, and the corresponding eigenvalues are as follows: It is evident that eig = (0.3959, 2.5000, 8000, 10.1041, 0, 0) and the rest are that the obtained quadratic form is equivalent to a positive definite matrix:

(21) $$\begin{array}{c} Q\left(K\right)=Q\left(K^{E}+K^{G}\right)=Q\left(K^{E}\right)+Q\left(K^{G}\right)>0. \end{array}$$

Demonstrating positive definiteness, it is established that the stability of the two-bar tensioned deployable mechanism is confirmed.

### 2.2. Model Building

#### 2.2.1. Three-Dimensional (3D) Model Building

**Figure 5:**
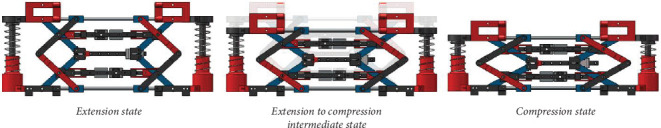
Model compression process.

Following the unit mapping and stability analysis of the two-bar tensioned integral spreadable mechanism, an establishment of a mechanism model is achieved for the two-bar tensioned integral spreadable mechanism. In practical applications, there is likewise the problem of excessive degrees of freedom, which requires constraints on the degrees of freedom of the model of the two-bar tensioning integral spreadable mechanism, as illustrated in Figure 5.

The deployable mast has two limit states, extension and retraction, and can attain a stable movement from 140 to 40 mm under the joint action of the spring and the connecting rod; in the extended state, the compression springs positioned at *AB* and *CD* on the left and right sides of the mechanism remain fully extended, the tension springs at *EG* and *FH* are not under stress, and the sliders on *AD* and *BC* are positioned at the extreme limits of the left and right ends. The angle between the connecting rods in this state reaches 90°, which enables the structure's stability in conjunction with compression spring rods *CD* and *AB*, and the overall height of the mechanism reaches its peak at this point. On the condition that the upper end of the mechanism is undergone by downward pressure, the compression springs on the left and right sides of the mechanism are pressed, and the lengths of *EG* and *FH* increase, resulting in the tension springs arranged here to elongate; the sliders at *AD* and *BC* slide toward the middle in a symmetrical attitude as well as drive the connecting rods into relative movement under the meshing action of the gear sets *C*_12_ and *C*_34_, leading to the angle between the connecting rods to decrease, thus decreasing the overall height of the mechanism; the transition from extension to retraction can be achieved. This reduces the overall height of the mechanism and enables a changeover from extension to retraction. When the mechanism requires transitioning to the extended state, the energy stored in the pressure springs *AB*, *CD*, *EG*, and *FH* on both sides is released, causing the slider to return to the limit position at the left and right ends under the force's influence. This action drives the connecting rod back to the extended state, facilitating the seamless transition between the two states.

To ensure the stability and symmetry of the structure during the change of state, the gears *C*_12_ and *C*_34_, which are arranged in the center of the mechanism, must be kept in mesh at all times and thus achieve a symmetrical rotation of the linkage concerning the central axis. To ensure the required degrees of freedom in the switching between the two states, a sliding subfit is incorporated along the mechanism's central axis, which reduces the influence of friction and limits the displacement error in the *y* direction, facilitating the deployable mast mechanism to attain a zero Poisson's ratio and stable extension with zero retractions in the *XOZ* plane.

#### 2.2.2. Prototype Build

**Figure 6:**
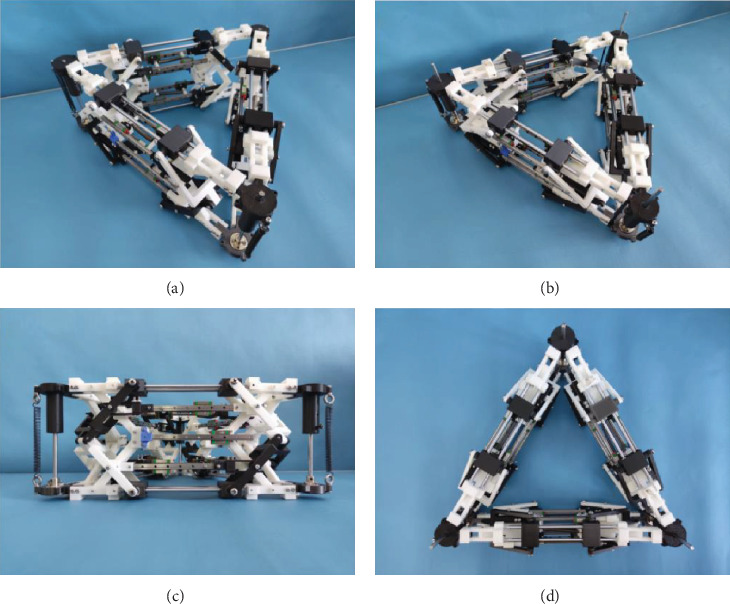
Diagram of the prototype. (a) Axonometric view of the unfolded state of a trigonal extension unit. (b) Axonometric view of the compression state of a trigonal extension unit. (c) Front view of the trigonal extension unit in its expanded state. (d) Top view of the trigonal extension unit in its expanded state.

The structure of the test prototype consists of numerous material compositions, specifically including the rod and part of the support parts utilizing photosensitive resin and PLA material for 3D printing processing; due to the hardness and smoothness requirements, the upper and lower metal connecting rods employed are made of 5-45# steel chrome-plated rods with a diameter of 5. The upper, lower, and middle sections of the slide utilize the standard MGN7C slide. The tension springs on the left and right sides have dimensions of 0.8 × 8 × 30 (wire diameter × outer diameter × length). The dimensions of the upper and lower springs are calculated as 0.8 × 8 × 30 (wire diameter × outer diameter × length). The inclined rods have a length of 80 mm, while the upper and lower rods measure 280 mm, and the left and right rods are 140 mm long. A spur gear with an external meshing configuration is employed, featuring a module of 1, tooth count of 15, inner diameter of 5 mm, outer diameter of 17 mm, and tooth thickness of 10 mm. The connection between the two external meshing gears is facilitated by MGN5 sliding rail combined with MGN5C sliding block. Specifically, the MGN5 slide has a length of 50 mm, width of 5 mm, and hole spacing at intervals of every15 mm, whereas the MGN5C slide measures a length of 17 mm, width of 12 mm, and hole spacing at in tervals of every 8 mm. (All slides and blocks used in the prototype are of this model.) The size of the whole machine at rest is 280 mm × 58 mm × 140 mm; in this design, it is compressed to the limit which is 280 mm × 58 mm × 90 mm; in accordance with the literature [42–44], the trigon extension arm has high stability. Lu et al. [45] indicate that the mechanism serves as the foundation for triangular prism design, with the traditional self-restoring deployable mechanism often integrated into it. However, this integrated forming mechanism has limitations in terms of load-bearing capacity and inability to be split. Therefore, incorporating tension into the deployable mechanism has emerged as a crucial direction. The tension mechanism is an assembly characterized by high hardness, offering both self-recovery and multilayer splitting capabilities. The prototype is demonstrated in Figure 6.

Figure 6a illustrates the stationary state, the spring of the mechanism is at rest, and the elastic potential energy is 0. In the process of diagram a to diagram d, with compression, the slider in the mechanism slides, the tension spring undergoes deformation, and the potential energy commences rising from 0; while the compression spring likewise undergoes deformation, the potential energy starts to increase from 0 on the condition that the compression reaches a height of 90 mm and the limit state is reached; at this time, the potential energy of the spring is maximum; at this time, the mechanism has the characteristics of two-rod tension: Self-recovery, in the limit state, can return itself to the initial state within an extremely brief time and can be very smooth and smooth to attain an extremely rapid transition from compression to expansion.

#### 2.2.3. Structural Characterization of the Deployable Mast Mechanism

**Figure 7:**
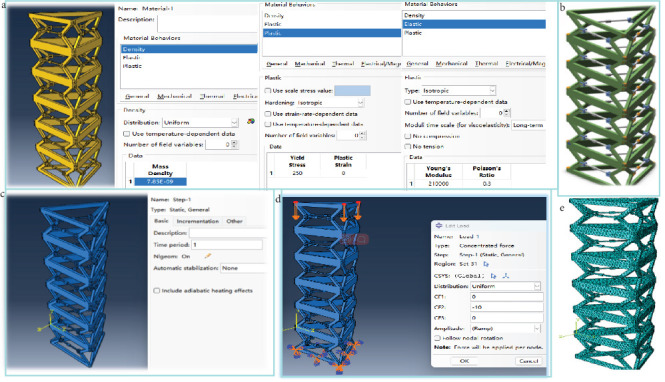
Analytical model of the deployable mast mechanism: (a) parameter determination, (b) deployable mast mechanism, (c) establish the static general analysis step, (d) applied load, and (e) mesh generation.

The key geometric parameters are as follows: the side length of the triangular section of the mechanism is 280 mm; the height of the (unit) mechanism in the fully expanded state is 140 mm; and the bars have a width and thickness of 15 mm and 5 mm, respectively. It has the following: 105 MPa, Poisson's ratio equal to 0.3, yield strength of 250 MPa (Figure 7a) [46–49]. In the first place, make the extension arm mechanism in a stable structural state, define the bottom end of the mechanism as a fixed-end constraint, and apply the load to the mechanism which can be analyzed accordingly; the analysis model of the deployable mast mechanism is illustrated in Figure 7b. Establish the static general analysis step, activate the geometric nonlinear switch, ensure displacement generation in the model, and define a time length of 1 with an incremental step size of 0.05. Refer to Figure 7c. Apply a concentrated downward force of 10 N on the upper surface of the component and impose fully fixed mechanical boundary conditions on the lower surface to maintain its current environmental position. As depicted in Figure 7d, assign grid control properties to the model components using 10-node quadric tetrahedral elements while excluding mapped triangular mesh usage on boundary surfaces for ensuring mesh integrity [49–52] (Figure 7e).

The coordinate origin is located at the vertex of the triangular section, the *y*-axis is adjacent to one side of the triangular section, and the *z*-axis is in the direction of extension of the deployable mast mechanism, perpendicular to the direction of the triangular section.

#### 2.2.4. Static Compression Stiffness Analysis

It is recognized that the deployable mast mechanism possesses a degree of freedom of 1. Consequently, locking one slider is sufficient to keep the mechanism in a stable structural state. Increasing the quantity of locked sliders undoubtedly results in an augmentation of the mechanism's stiffness. The middle slide on the same face is locked in this way, and the static compression stiffness of the mechanism is analyzed for three distinct slide-locking cases: (a) one slide locked, (b) two slides locked, and (c) three slides locked.

**Figure 8:**
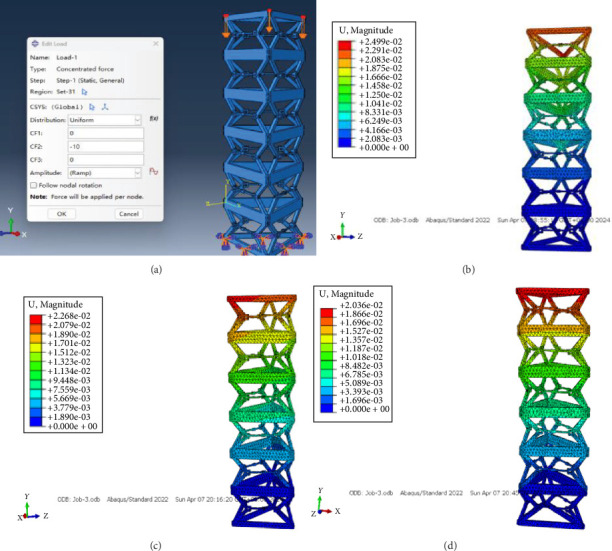
Load action diagram and maximum displacement of the mechanism nodes for different locking cases: (a) load action diagram, (b) locking a slide, (c) locking both slides, and (d) locking three slides.

A force of 10 N was applied to each of the three hinge points at the top of the deployable mast in the direction perpendicular to the triangular section. Besides, the action diagram of the load acting on the analytical model and the results of the analysis for distinct locking cases are demonstrated in Figure 8.

The relationship between the deformation of a structure under load and its static stiffness is *K*_C_ = *F*_P_/*δ*; in the formula, *K*_C_ denotes the compression stiffness of the mechanism, and *δ* illustrates the displacement of the end of the mechanism under load force.  Table 3 displays the static compression stiffness of the mechanism across various locking scenarios.

As evident from the table provided, the compression stiffness of the mechanism is hardly dissimilar overall, and yet it appears from the data that the locking three pairs of sliders possess optimal compression stiffness.

#### 2.2.5. Inherent Frequency Analysis

The above model was employed to analyze the split inherent frequency of the deployable mast mechanism in divergent locking states, and the results of this analysis are demonstrated in Table 4. The table results indicate that the natural frequency of the deployable mast mechanism remains relatively stable, and variations in the logarithm of the slider's locking have minimal impact on the inherent frequency of the structure. In combination with the static compression stiffness analysis of the structure, to ensure the best compression stiffness of the mechanism and at the same time to guarantee that the mechanism can be compressed with a balanced force, the deployable mast mechanism is put into working condition by locking three pairs of sliders.

#### 2.2.6. Conclusion

Based on ABAQUS (DS, France) finite element analysis software of the extension arm mechanism and the structure performance of the statistics, the minimum compression stiffness of the space extension arm of the extension arm is 9 × 105 N/m; the first-order vibration frequency should be greater than 1 HZs; so from the analysis results, the extension arm mechanism can be applied in engineering practice.

### 2.3. Simulation Incorporating Tensioning Properties

**Figure 9:**
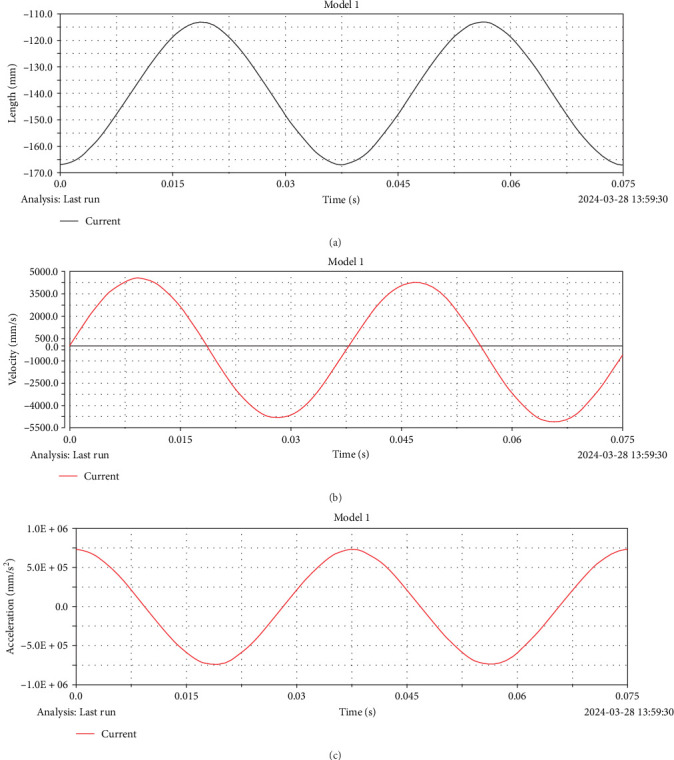
(a) Time–displacement simulation with tensile characteristics, (b) velocity–displacement simulation with tensile characteristics, and (c) acceleration–displacement simulation with tensile characteristics.

By importing the 3D model into ADAMS (MSC, USA) for dynamic simulation, the relationship curves of displacement *x*, velocity *v*, acceleration *a*, and time *t* of the rod in the *z*-axis are likewise obtained.  Figure 9a–c illustrates the temporal evolution of the displacement, velocity, and angular acceleration curves at the *z*-axis endpoint of the rod.

### 2.4. Tradition Simulation

**Figure 10:**
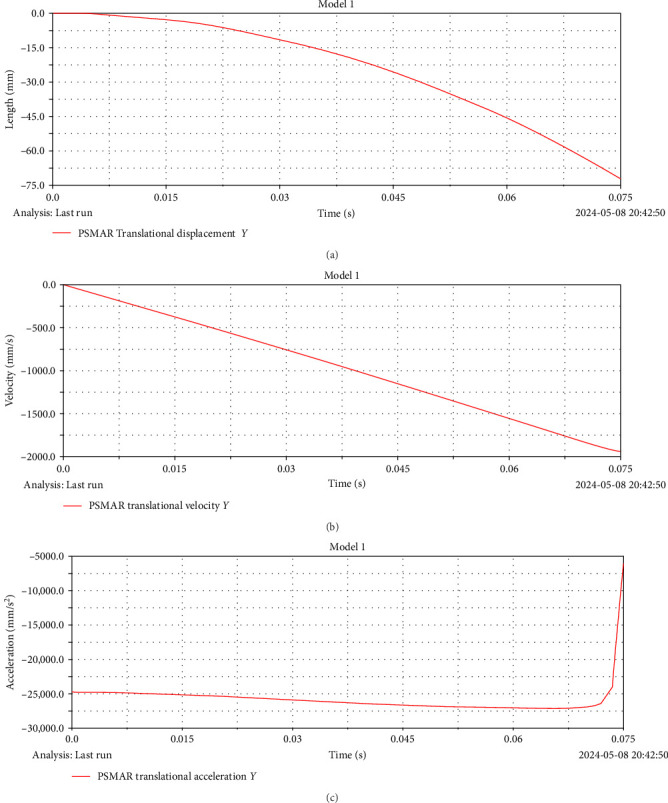
(a) Time–displacement simulation with traditional structure, (b) velocity–displacement simulation with traditional structure, and (c) acceleration–displacement simulation with traditional structure.

The traditional exhibition mechanism without tension characteristics is introduced into ADAMS (MSC, USA); the relationship curves of displacement *x*, velocity *v*, acceleration *a*, and time *t* of the rod in the *z*-axis are likewise obtained.  Figure 10a–c illustrates the temporal evolution of the displacement, velocity, and angular acceleration curves at the *z*-axis endpoint of the rod.

It can be observed from Figure 9 that the displacement *x*, velocity *v*, and acceleration *a* all show a trend of periodic reciprocating motion in a full period. This phenomenon further verifies that the mechanism has self-recovery and shows its feasibility and stability in practical application. Meanwhile, compared with the traditional exhibition mechanism of Figure 10, the traditional exhibition mechanism is not self-recovery and does not have periodic reciprocating movement in the process of movement. Following confirming that the mechanism model can achieve the motion required by the single-layer expansion mechanism, we additionally validate the mechanism's ability to recover itself from the compressed state to the expanded state. Through deep practical calculation and simulation tests, we comprehensively evaluate the motion performance of the deployable mechanism and fine-tune it to satisfy the functional design specifications in place of the whole deployable unit. This intricate and meticulous process comprehensively guarantees the academic credibility and engineering applicability of the research.

### 2.5. Experiment

#### 2.5.1. Force and Displacement Experiments

**Figure 11:**
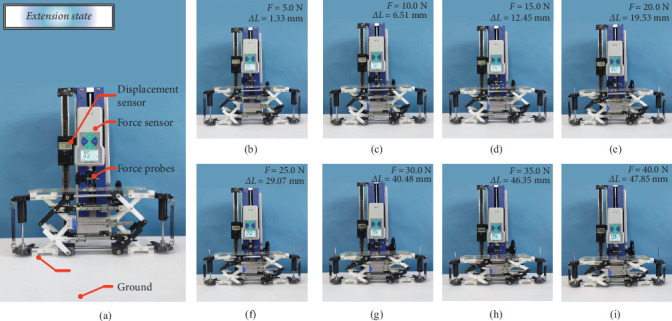
Experimental force test diagram of the stability of the mechanism. (a) Experimental bench and test prototype and (b–i) load experimental measurement data.

For the purpose of proving that the proposed deployable mast mechanism has self-recovery characteristics while achieving fast extension and can substantially enhance the efficiency of the deployable mast, we adopted high-precision distance sensors and pressure sensors to test the relationship between the magnitude of the force on the deployable mast unit as well as the displacement in the vertical direction; the experimental bench and test prototype are displayed in Figure 11a,b,i which demonstrate the seven sets of experimental data measured.

Several sets of measurements were extracted from 0 to 40 N to demonstrate the self-recovery stability and rapid deployment characteristics of the tensioned integral spreadable mechanism.

**Figure 12:**
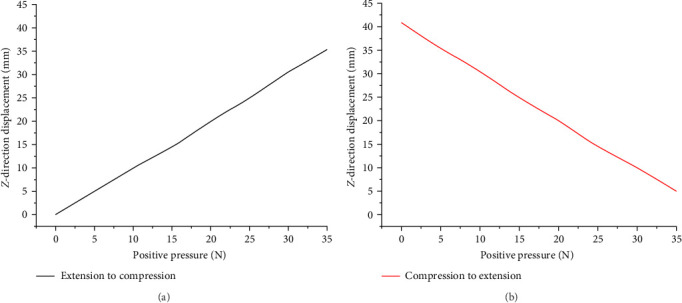
Positive pressure versus displacement curve. (a) Extension to compression and (b) compression to extension.

By exhaustively collecting and analyzing several sets of data, we plotted the relationship curve between positive pressure and displacement. The table in Figure 12 shows the relationship between the mechanism units in the compressed and stretched states. Figure 12a illustrates the trend of the mechanism unit transitioning from the extended state to the compressed state. Figure 12b demonstrates the trend of the mechanism unit transitioning from the compressed state to the extended state. Based on the comprehensive analysis of the experimental data, we conclude that the observed displacement–force relationships for both states show a clear linear increase. This experimental result further accentuates the consistency of the behavior of the mechanism unit during unfolding to compression and compression to unfolding, demonstrating a stable and predictable linear trend in its mechanical response. Analyzing the curve characteristics in-depth, we observe a linear increase in displacement versus force for both the unfolding to compression process and the compression to unfolding process of the mechanism unit, which strongly indicates the high stability and low error characteristics of the tensioned integral extension arm. This pattern aligns with the stability that is necessary throughout the tensioning process, including the compression and unfolding stages. This further illustrates its exceptional performance as the spatial extension arm extends. Moreover, this linearly increasing relationship curve provides insight into the behavior of the mechanism unit as well as provides a sound theoretical basis for superior performance in design. Overall, the results acquired not only showcase the reliability and stability of the mechanism unit but also underscore its excellence in fulfilling the spatial extension arm's extension requirements. The phenomenon of linear growth offers insights into the performance of the mechanism unit from an experimental perspective and establishes a dependable foundation for future design and engineering applications.

#### 2.5.2. Quasistatic Compression-Unloading Experiment

**Figure 13:**
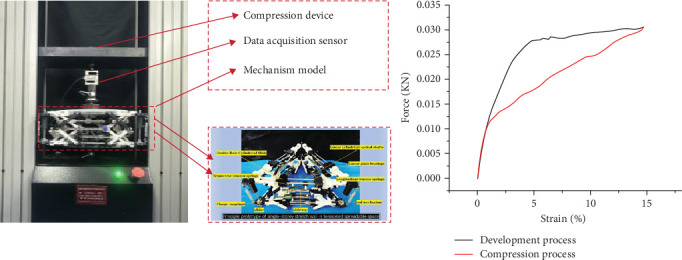
Compression–unloading test of universal testing machine.

To reveal the force–strain response of the mechanism during unfolding and compression, we tested the 3D-printed test specimens by testing them on a Zwick-Roell Z050 universal testing machine.  Figure 13 indicates the universal testing machine composition mechanism. Moreover, the tests were conducted utilizing a strain rate of 0.33 mm/s up to 100% of the total strain to gain insight into the unfolding and compression behavior of the mechanism under dynamic loading (Supporting Information 2).

Quasistatic compression-unloading experiments were conducted on the structure using a universal testing machine to gather data and generate the curves illustrated in, where the red curve is the force–strain response during loading and the black curve is the force–strain response during unloading. Furthermore, the experimental results indicate that during the test, the mechanism was displaced by 25 mm in the range of load *F* (0–35 N), and following the release of the load, the mechanism was able to autonomously recover to the initial state without external forces (the experimental procedure is available in Supporting Information 1). It is worth noting that the trajectories of the mechanism in unfolding to compression and compression to unfolding are symmetrical, and the stress–strain curves create closed intervals [37]. This interval proves the energy storage property of the elastic member inside the tension body; that is, the elastic body shortens the energy storage during compression and springs up automatically on the condition that the external load is withdrawn, in the absence of further coercion to reinstate the structure to its original configuration, which recapitulates the self-recovery property of the tension body in the design of mechanical structures.

## 3. Conclusions

In this paper, through the in-depth study of the function of the extension arm mechanism, an innovative extension arm structure is proposed, which adopts the design concept on the basis of the two-bar tension integral extendable mechanism. Moreover, the introduction of the tensioning machine structure gives the extension arm self-recovering as well as self-stabilizing characteristics, and this design change has modified the traditional motor-driven method of the space extension arm. The results were *Q* (*k*) > 0, which verified the characteristics of high stability. Additionally, the static stiffness and intrinsic frequency of the extension arm mechanism are analyzed in detail by ABAQUS (DS, France) finite element analysis software, and the structural performance statistics of the aerospace extension arm mechanism applied in real engineering are also conducted. The results demonstrate that the extended arm mechanism has sufficient minimum compressive stiffness (9 × 10^5^ N/m) and first-order vibration frequency greater than 1 Hz to be applied in practical engineering. In terms of kinetic simulation, it can be seen from the figure that the curve of displacement and time is a curve of smooth reciprocating motion, the reciprocating period is 0.0375 s, the maximum value is−113 mm, and the minimum value is−167 mm. The velocity and time curves are sinusoidal function curves with a reciprocating period of 0.0375 s and a minor value of 5000 mm/s. The acceleration and time curves are cosine function curves, with a reciprocating period of 0.0375 s and a minor value of 750,000 mm/s^2^. The data show that there is a reciprocal movement during the mechanism removement, proving its self-recovery nature. To verify that the designed extension arm mechanism has tension self-recovery characteristics and high stability, force–displacement experiments and force–strain experiments were conducted. In the process of compression to unfolding, the force and displacement exhibit a linear increase, whereas the force and strain tend to conform to a closed curve. Through the high-precision distance sensor and the pressure sensor, the relationship between the force size and the displacement of the vertical direction is tested. When the force is gradually increased from 0 to 40 N and the step size is 5 N, the vertical displacement *∆L* is gradually increased from 1.33 to 47.85 mm. The self-recovery stability and rapid unfolding properties of the mechanism are demonstrated. The stress–strain curve of the universal tester rises steadily between 0 and 30 N under the action of compression force between 0 and 35 N and gradually stabilizes after 30 N with no obvious change. In the unloading state, the stress drops from 35 to 12 N, and the stress decreases steadily and gradually stabilizes to 0 with no significant change. It proves the energy storage characteristics of the internal elastic member of the pulled body; that is, in the case of external load withdrawal, the elastic member shortens the energy storage and automatically bounce during the compression process, without further forcing the structure to restore the structure to the original form, which reproduces the self-recovery characteristics of the pulled body in the mechanical structure design. A detailed analysis of the experimental data concludes that the mechanism exhibits excellent stability and remarkable self-recovery in the process. This relationship between linear displacement and force, as well as the characteristic of a closed curve between force and strain, provides an observable physical basis for the design of the mechanism and provides a strong support for its working performance in practical applications. By reducing the requirement for unfolding to compression drive motors, the mechanism effectively alleviates the strain on the space unfolding mechanism.

Subsequent research endeavors will focus more on enhancing the unfolding speed and positional accuracy of the extension arm mechanism to obtain increased versatility in the unfolding process while reducing loads. The intensive exploration in this direction is anticipated to yield more advanced and dependable solutions for engineering applications in aerospace and other domain.

## Figures and Tables

**Table 1:** Deployable mast dimensional parameters.

*AB*	*AD*	∠*CFJ*	∠*FMB*
140	280	45°−15°	45°−75°

**Table 2:** Node association matrix of the deployable mechanism of the two-bar tensioned whole.

Element/node	*C*			
	1	2	3	4
*AB*	1	−1	0	0
*BC* (*FH*)	1	0	0	−1
*CD*	0	0	1	−1
*AD* (*EG*)	0	1	−1	0
*AC*	1	0	−1	0
*BD*	0	1	0	−1

**Table 3:** Static compression stiffness of the mechanism for different locking cases.

Institutional lock-in	Static compression stiffness (N/m)
Locking a slide	9.05515 × 10^5^
Locking both slides	9.40917 × 10^5^
Locking three slides	9.91159 × 10^5^

**Table 4:** Intrinsic frequency of the mechanism for different locking cases.

Institutional lock-in	Inherent frequency (Hz)					
	1	2	3	4	5	6
Locking a slide	302.08	317.66	318.81	328.54	329.25	339.54
Locking both slides	304.01	319.69	320.85	331.28	332.00	342.35
Locking three slides	305.98	321.76	322.93	334.07	334.79	345.22

## Data Availability

All data are available in the main text or the Supporting Information.

## Supporting Information

Supporting Information 1 Single-layer extension arm self-recovery experiment.

Supporting Information 2 Additional clarification and detailed information.
