# Supplementary material for: The Extension Arm Design Method Based on a Two-Bar Tension Stretchable Mechanism
Source: Appl Bionics Biomech. 2025 Feb 22;2025:3313533. doi: 10.1155/abb/3313533 (PMC11871976; doi:10.1155/abb/3313533)
Supplement: Supporting Information 1 — Single-layer extension arm self-recovery experiment. [file 3313533.f1.docx]

**Supplementary materials offer additional clarification and detailed information to support the main content：**

The exhibit presented here is a mechanical prototype device, specifically labeled as the "Principle Prototype of a Single-Layer Tensile Deployable Spatial Mechanism," designed to verify and demonstrate specific mechanical properties. Each component in the diagram is accompanied by detailed English annotations to clarify its function and role:

- Double Hole Cylindrical Slide: Acts as a crucial element for connection and support, ensuring precise movement paths of the mechanism.
- Linear Cylindrical Optical Shafts: Provides guidance for linear motion, ensuring smooth and accurate relative motion between components.
- Linear Plain Bearings: Reduces friction, facilitating smooth movement of sliders and serving as the cornerstone for efficient mechanism operation.
- Transverse Tension Springs and Longitudinal Tension Springs: Collectively constitute the elastic recovery system of the mechanism, responsible for maintaining and restoring structural balance.
- Flange Couplings: Enables the transmission of power or torque, ensuring coordinated motion among internal components of the mechanism.
- Slider: Acts as a moving part, responding to external forces to achieve the deployment and retraction of the mechanism.
- Sideway: Provides a clear motion trajectory for the slider, ensuring stability in the mechanism's motion.

The core focus of this supporting material lies in validating the self-recovery performance of the mechanism. In its initial static state, both the transverse and longitudinal tension springs are pre-tensioned, forming a self-balancing mechanical system. The diagram indicates an external force "F" acting on the upper platform, with arrows showing the direction of the force.

(a) State Analysis: Prior to the application of an external force, the mechanism maintains its initial equilibrium state, with the deformation states of the transverse and longitudinal springs ensuring overall stability.

(b) Dynamic Response Analysis: When an external force F acts on the upper platform in a specified direction, the platform undergoes downward displacement. During this process, the longitudinal spring is compressed, while the transverse spring is stretched, disrupting the original equilibrium state of the mechanism. As the external force F continues to be applied, the platform descends continuously to a certain critical position. Upon instantaneous removal of the external force F, due to the inherent self-balancing and self-stabilizing characteristics of the tensile mechanism, the platform immediately initiates the recovery mechanism. The transverse spring releases energy, while the longitudinal spring extends, driving the platform to rapidly return to its initial equilibrium state.

The essence of this phenomenon lies in the design of the tensile mechanism as a self-stabilizing system with intrinsic mechanical balance. Although an external force can temporarily disrupt this balance, causing deformation, once the external force disappears, the mechanism rapidly returns to its original state due to its internal elastic elements, demonstrating remarkable self-recovery capabilities. This process not only highlights the ingenuity of the mechanism's design but also provides important theoretical and practical foundations for research on deployable spatial structures.
