# Supplementary material for: The Extension Arm Design Method Based on a Two-Bar Tension Stretchable Mechanism
Source: Appl Bionics Biomech. 2025 Feb 22;2025:3313533. doi: 10.1155/abb/3313533 (PMC11871976; doi:10.1155/abb/3313533)
Supplement: Supporting Information 2 — Additional clarification and detailed information. [file 3313533.f2.pdf]

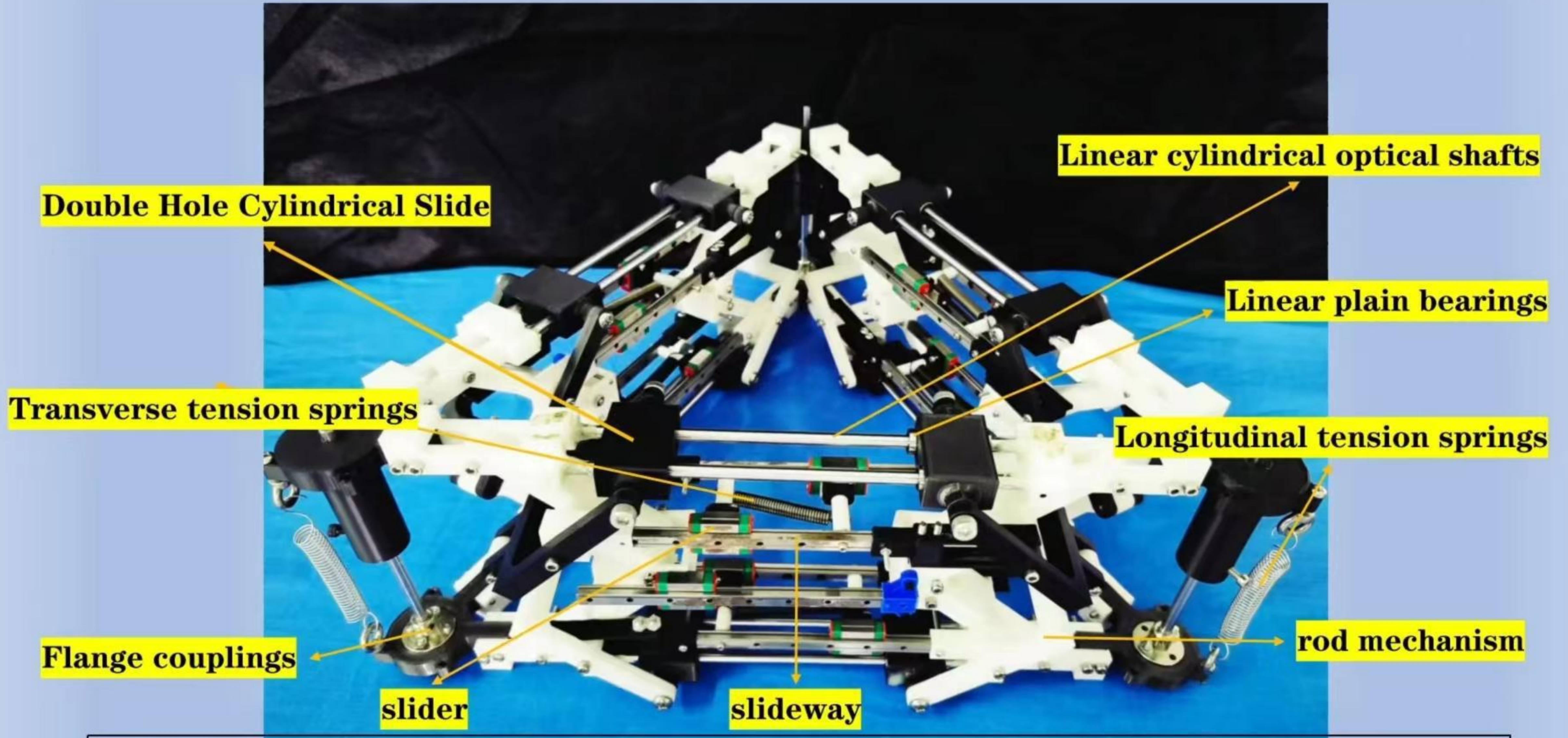

Principle prototype of single-storey stretch wall in tensioned spreadable space

➢ Test

Single-layer extension arm self-recovery experiment

(a) (b)

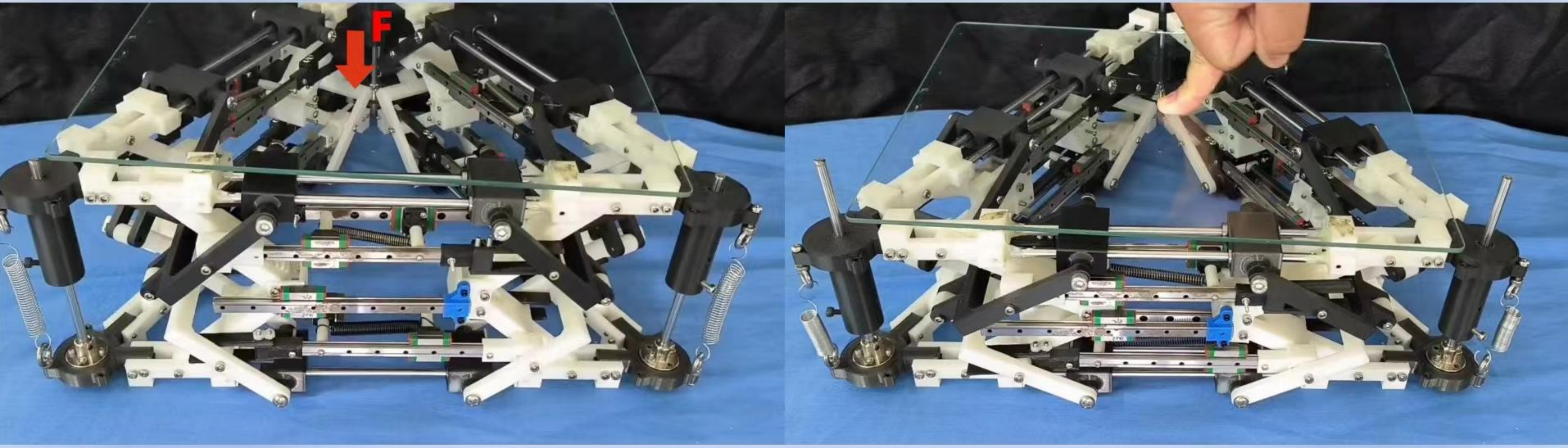

An external force F is applied to the upper platform in the direction shown by the arrow F. The upper platform moves downwards and reaches the critical compression position

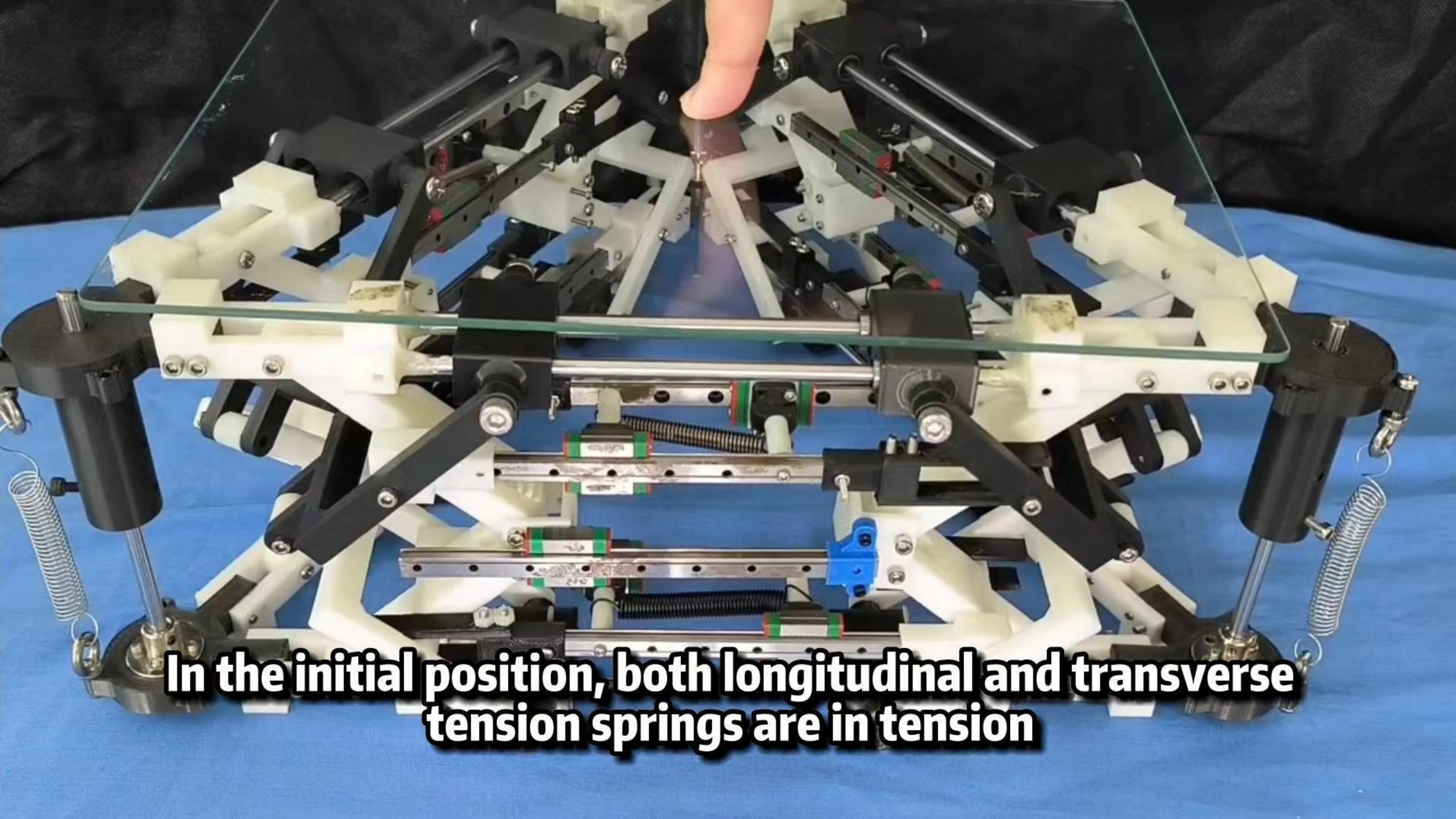
In the initial position, both longitudinal and transverse tension springs are in tension

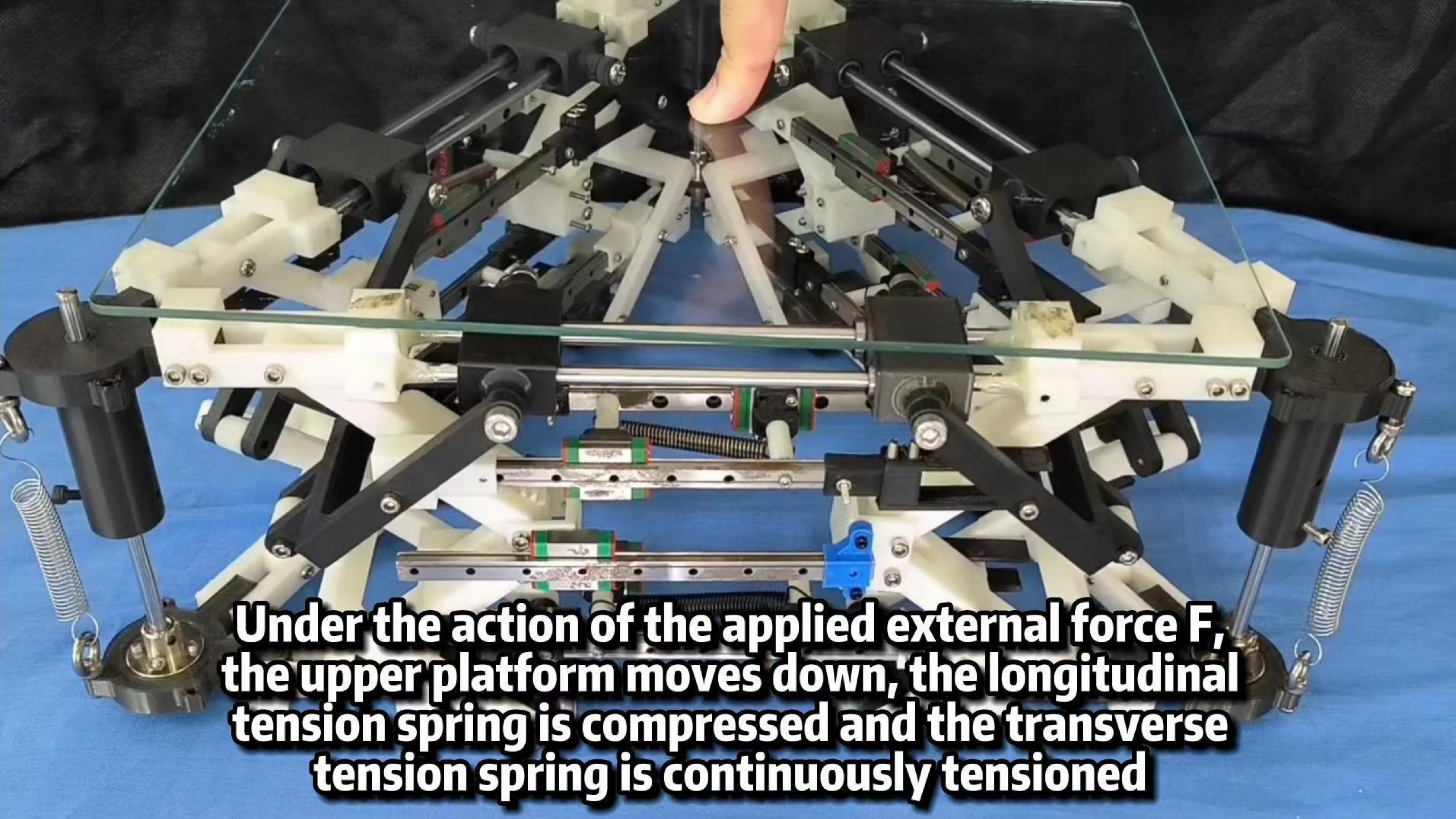
Under the action of the applied external force F,
the upper platform moves down, the longitudinal
tension spring is compressed and the transverse
tension spring is continuously tensioned

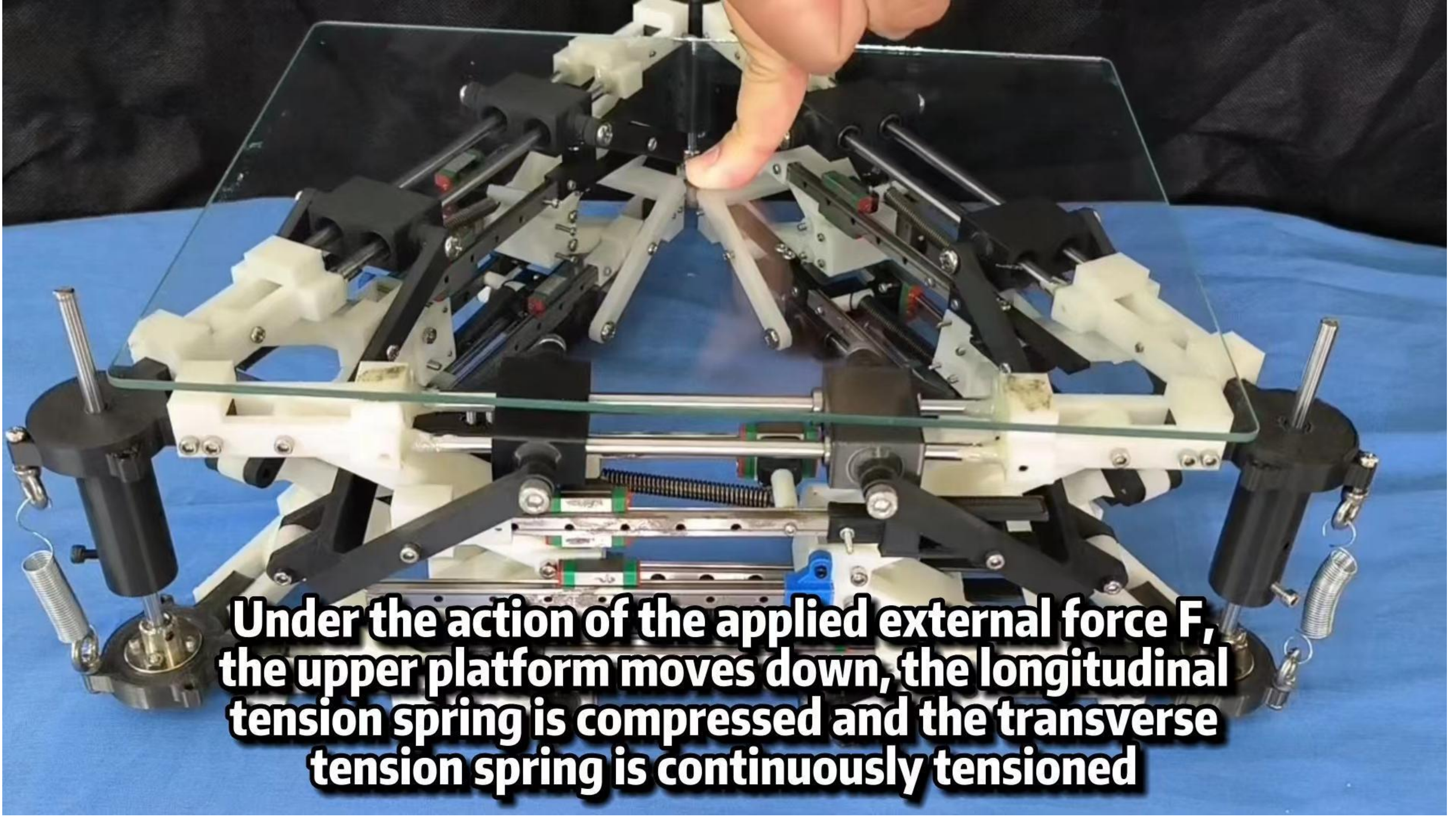
Under the action of the applied external force F, the upper platform moves down, the longitudinal tension spring is compressed and the transverse tension spring is continuously tensioned

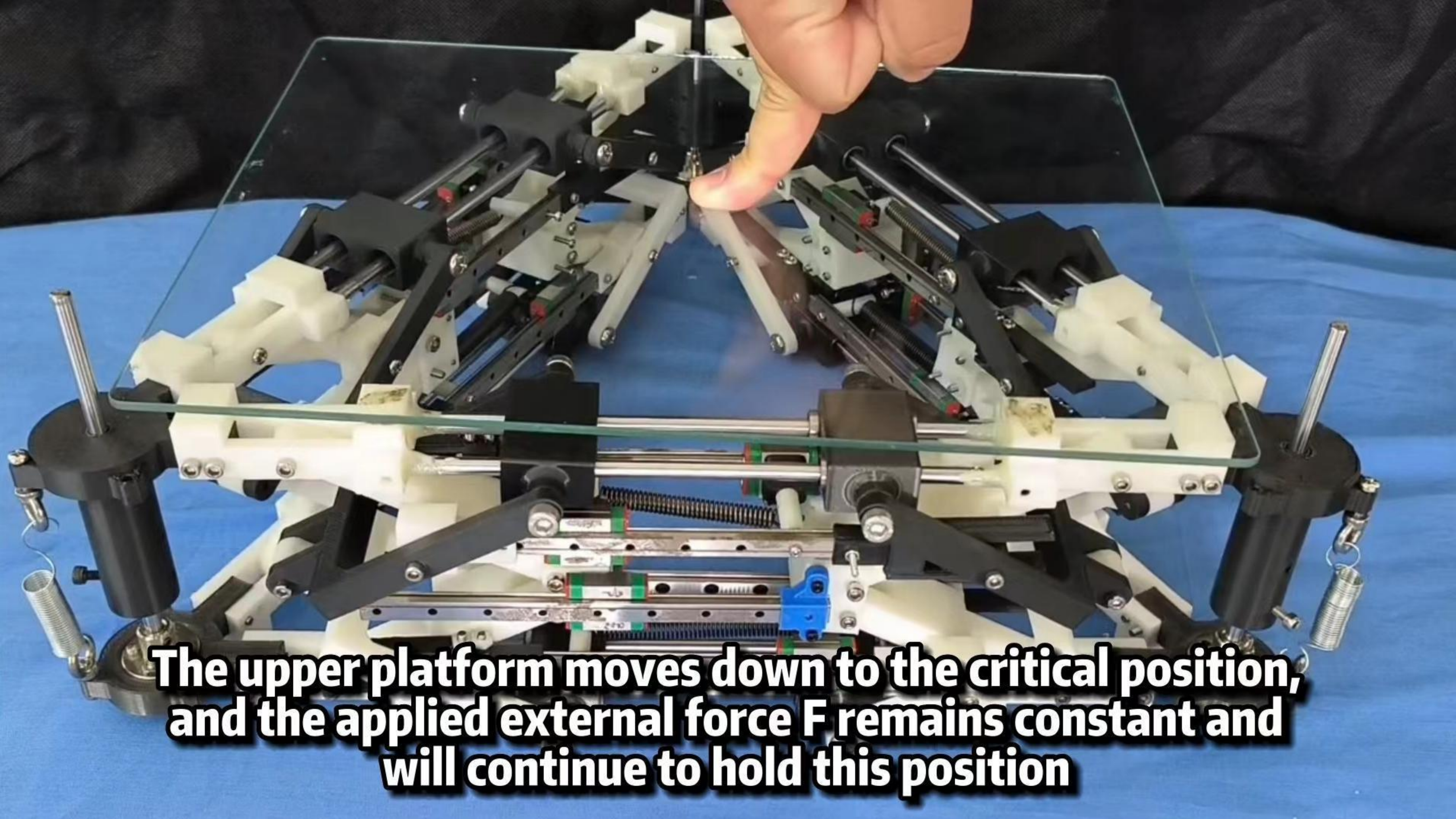
The upper platform moves down to the critical position,
and the applied external force F remains constant and
will continue to hold this position

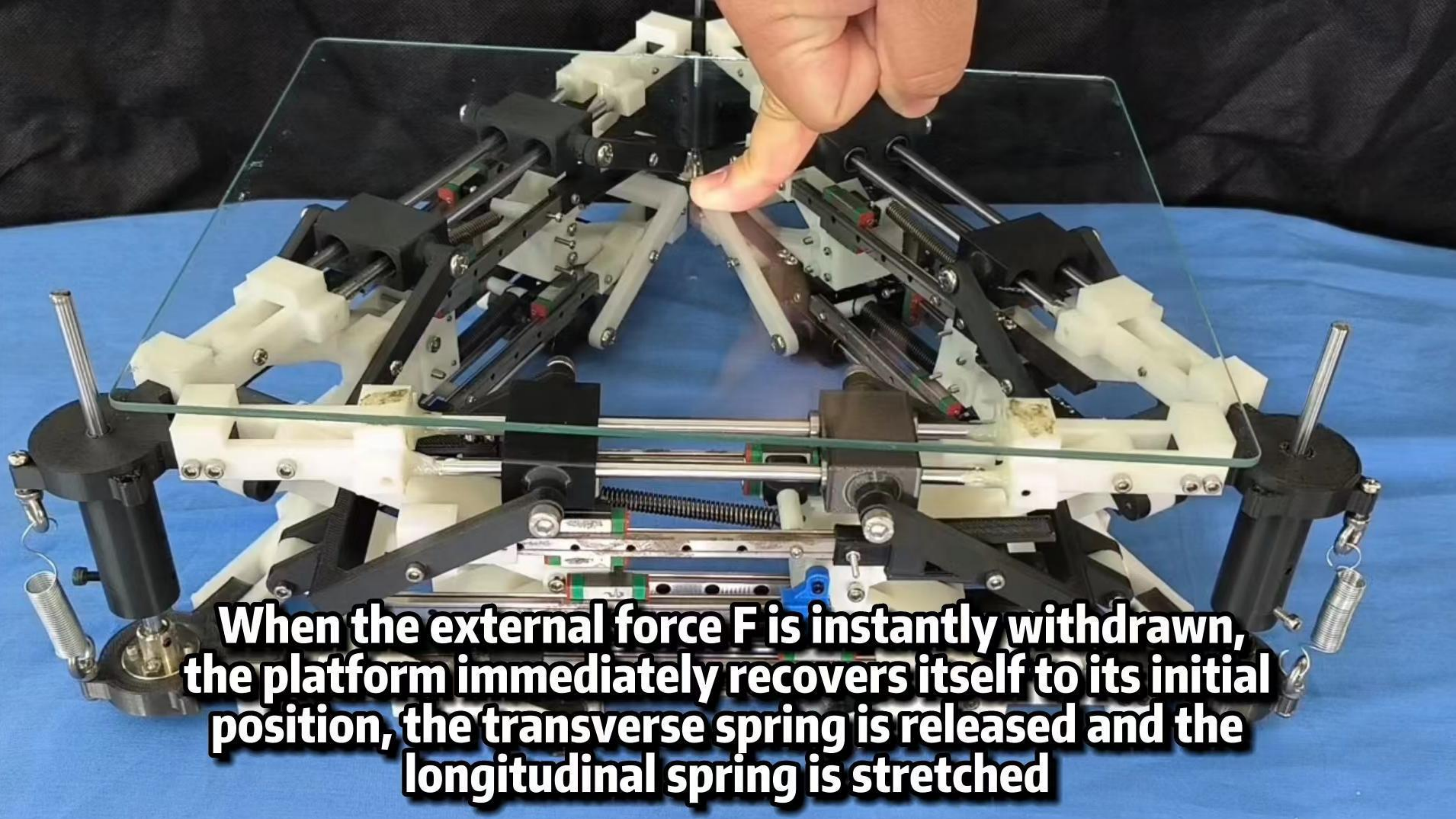
When the external force F is instantly withdrawn,
the platform immediately recovers itself to its initial
position, the transverse spring is released and the
longitudinal spring is stretched

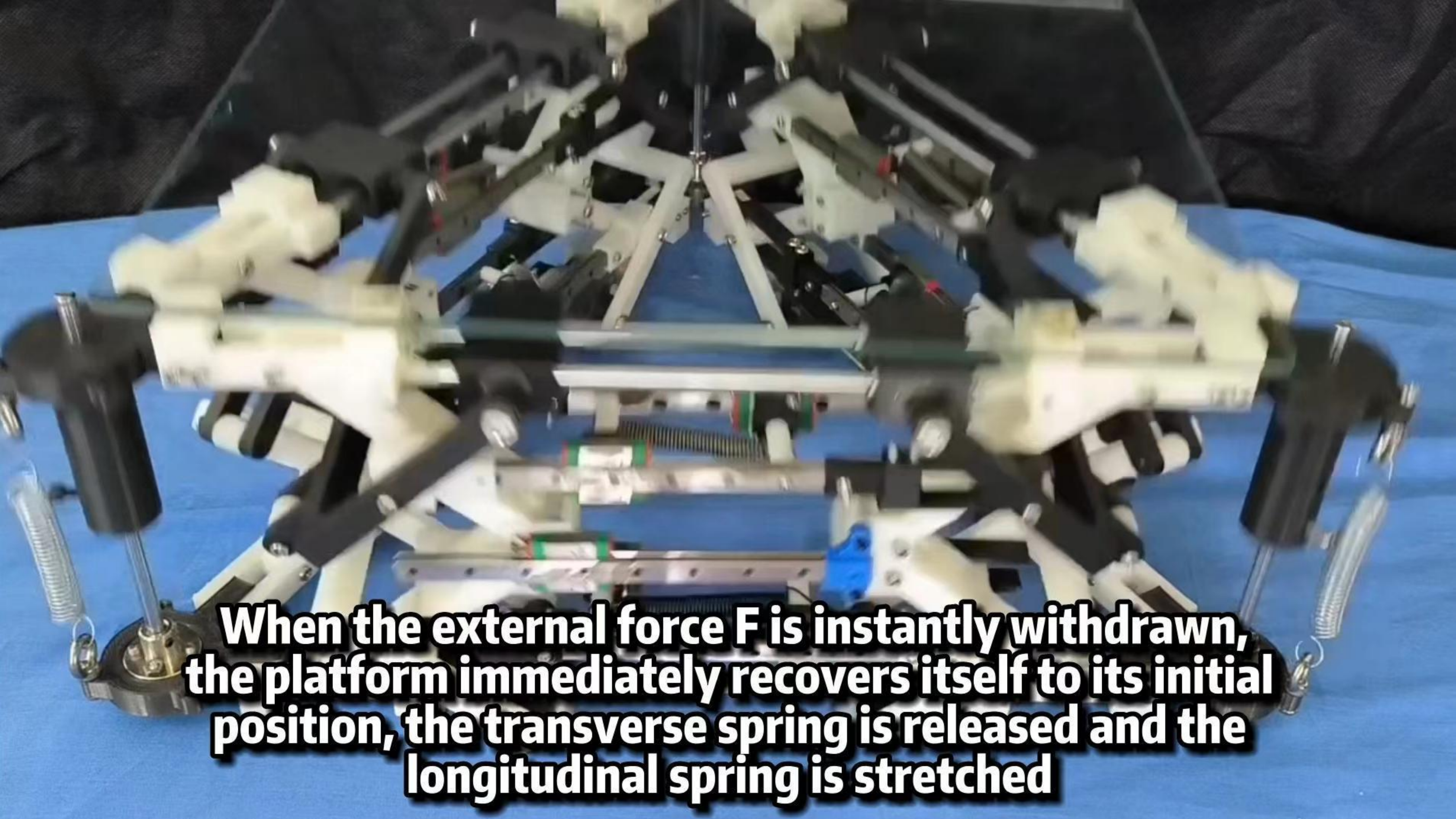
When the external force F is instantly withdrawn,
the platform immediately recovers itself to its initial
position, the transverse spring is released and the
longitudinal spring is stretched

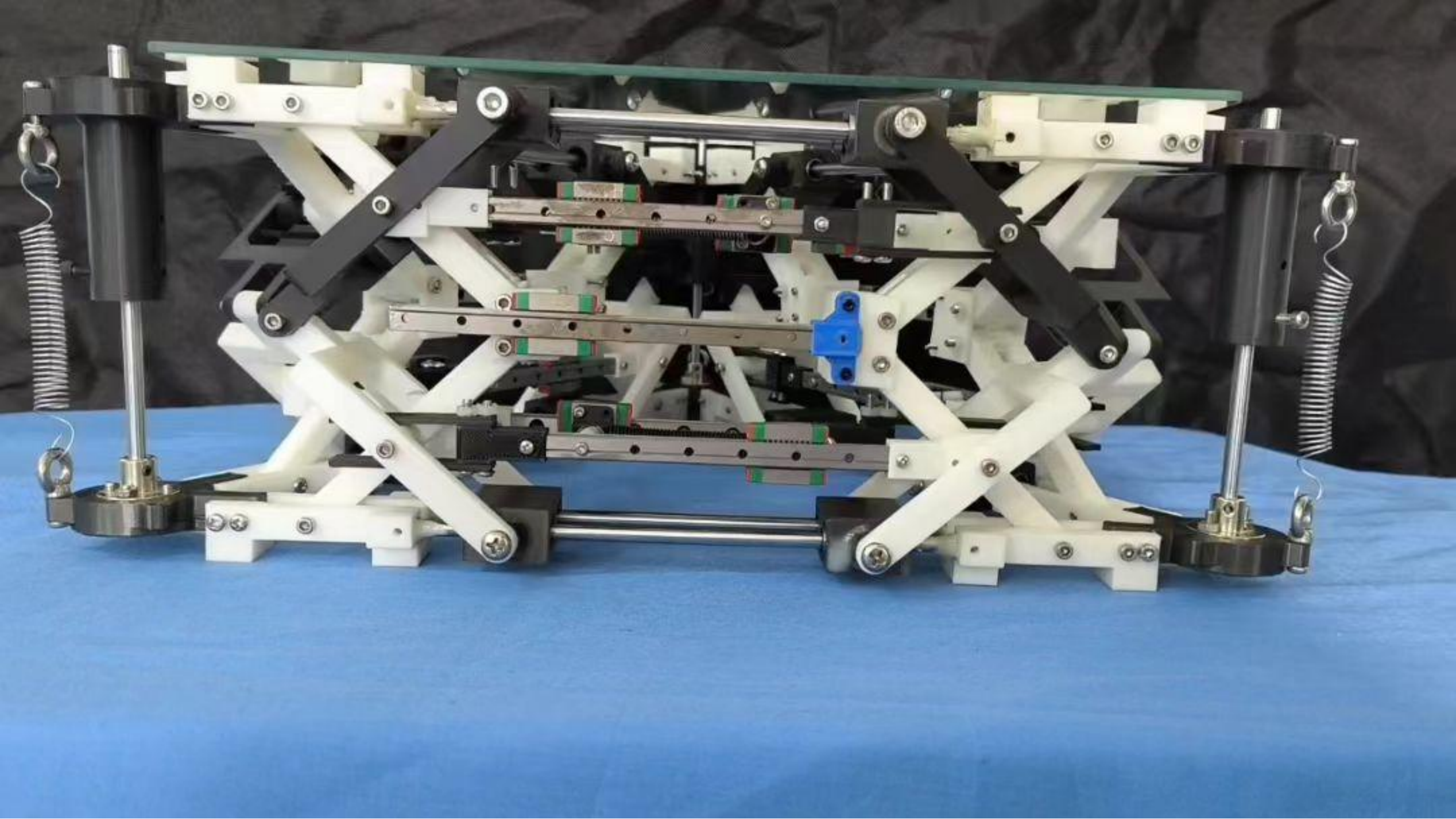

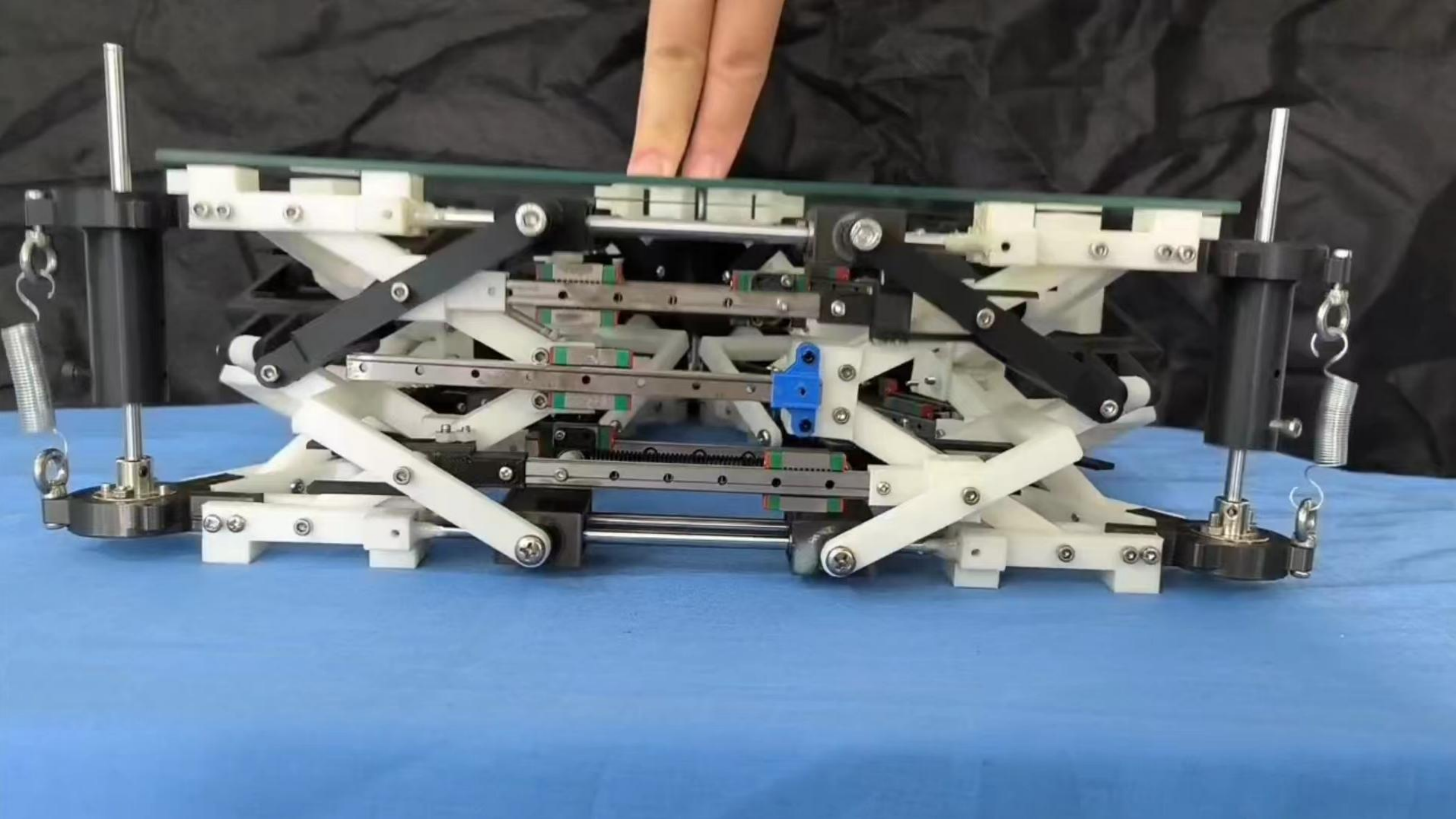

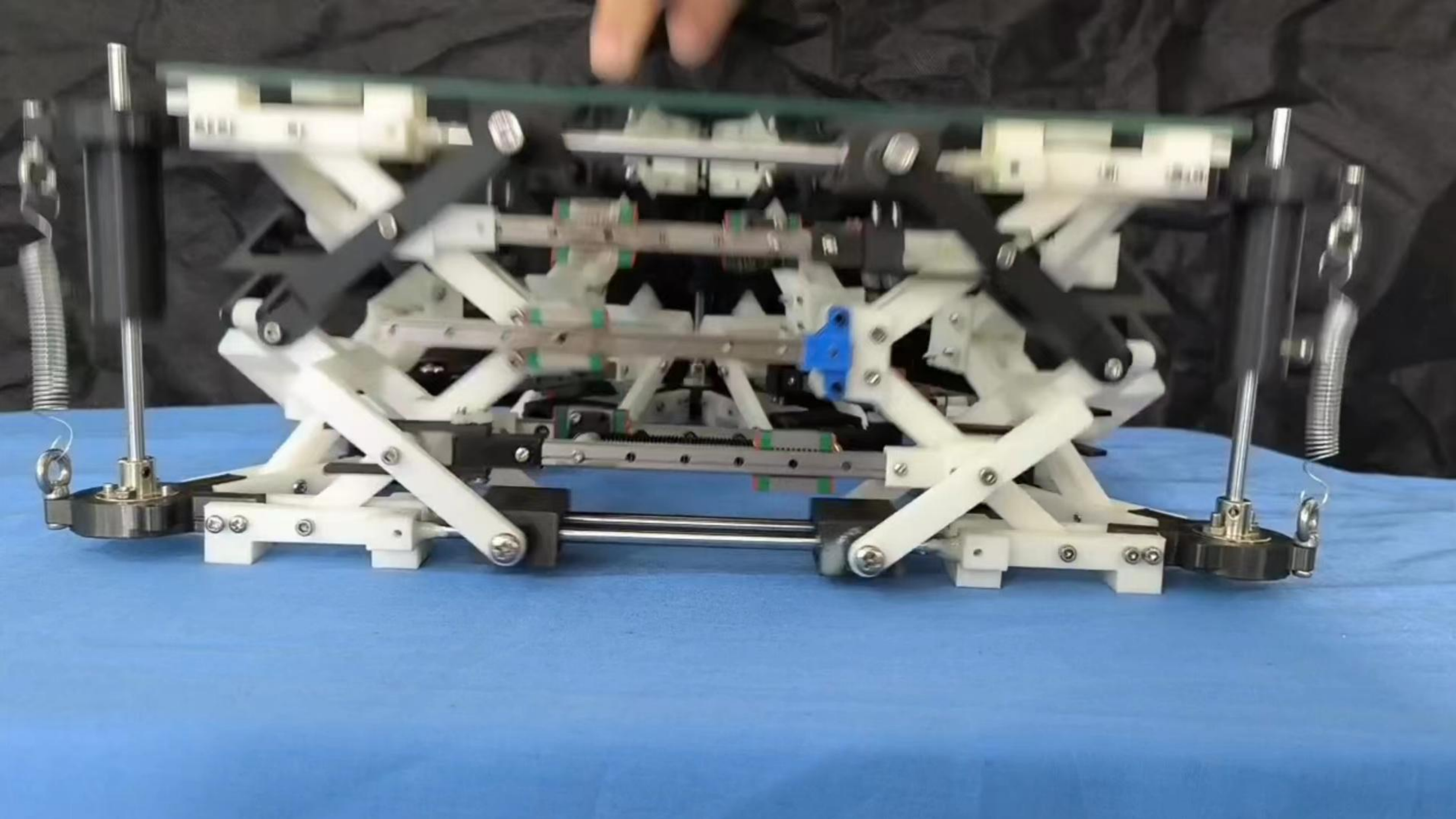

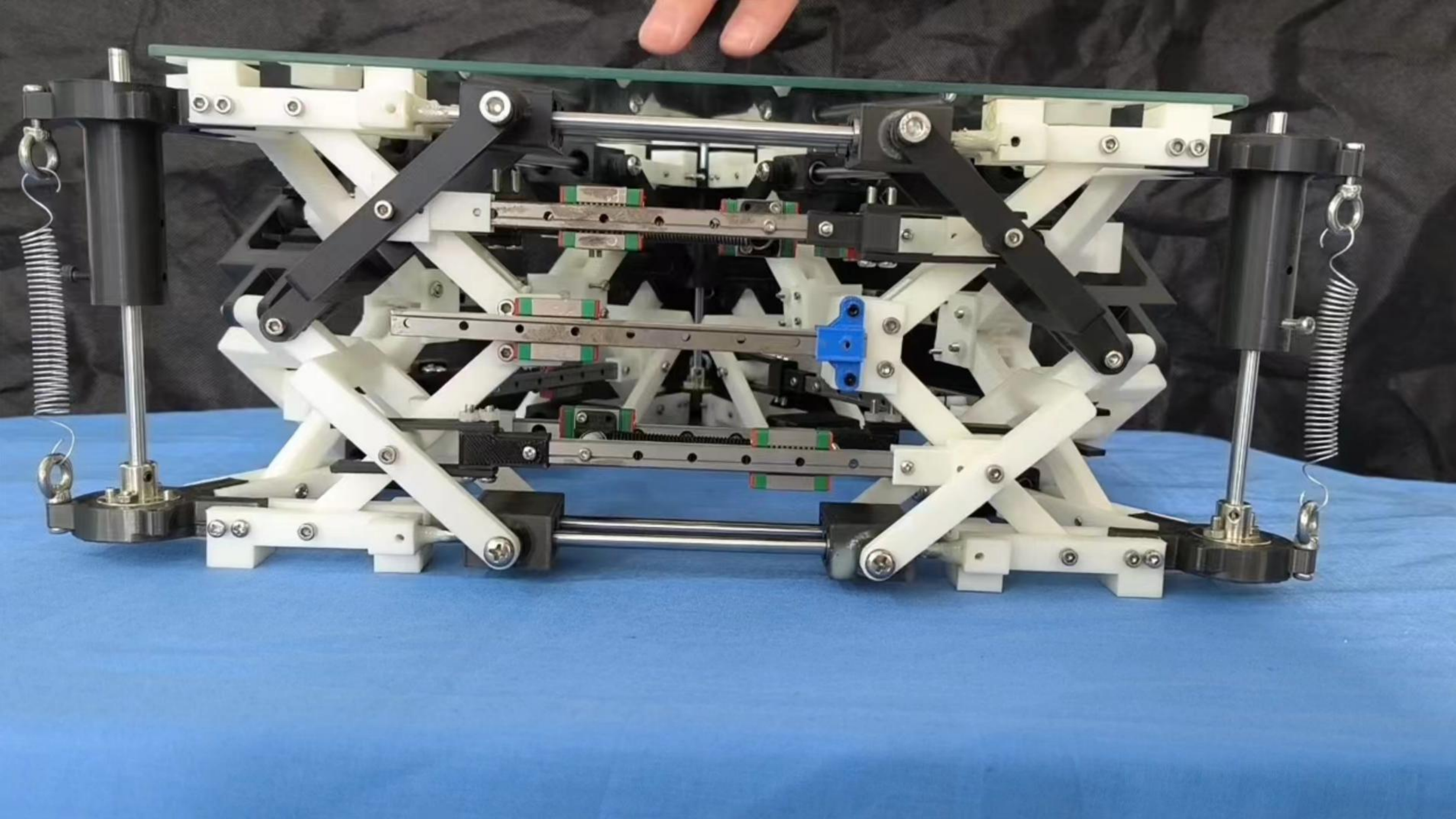

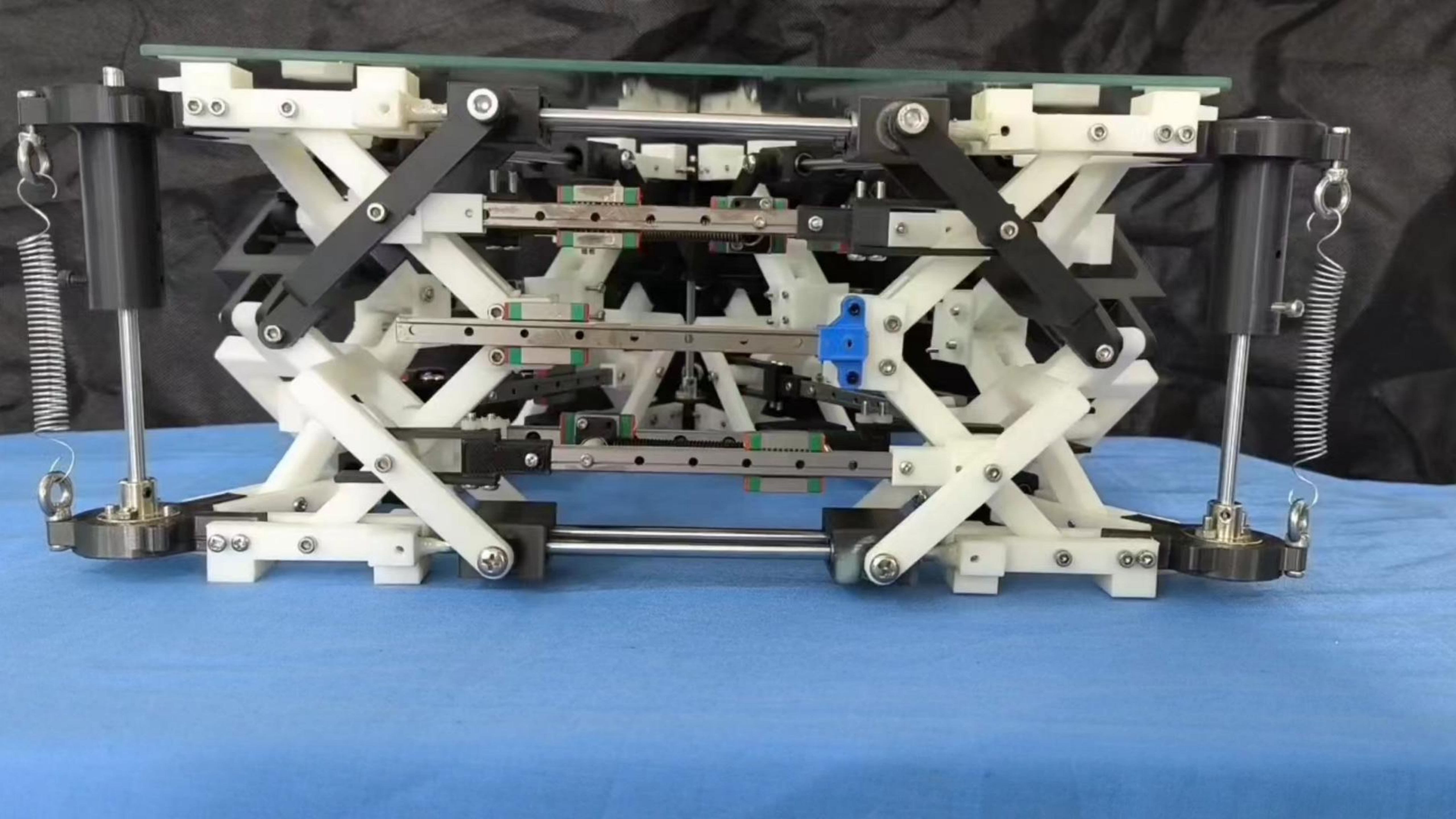

Playback Speed
0.5x

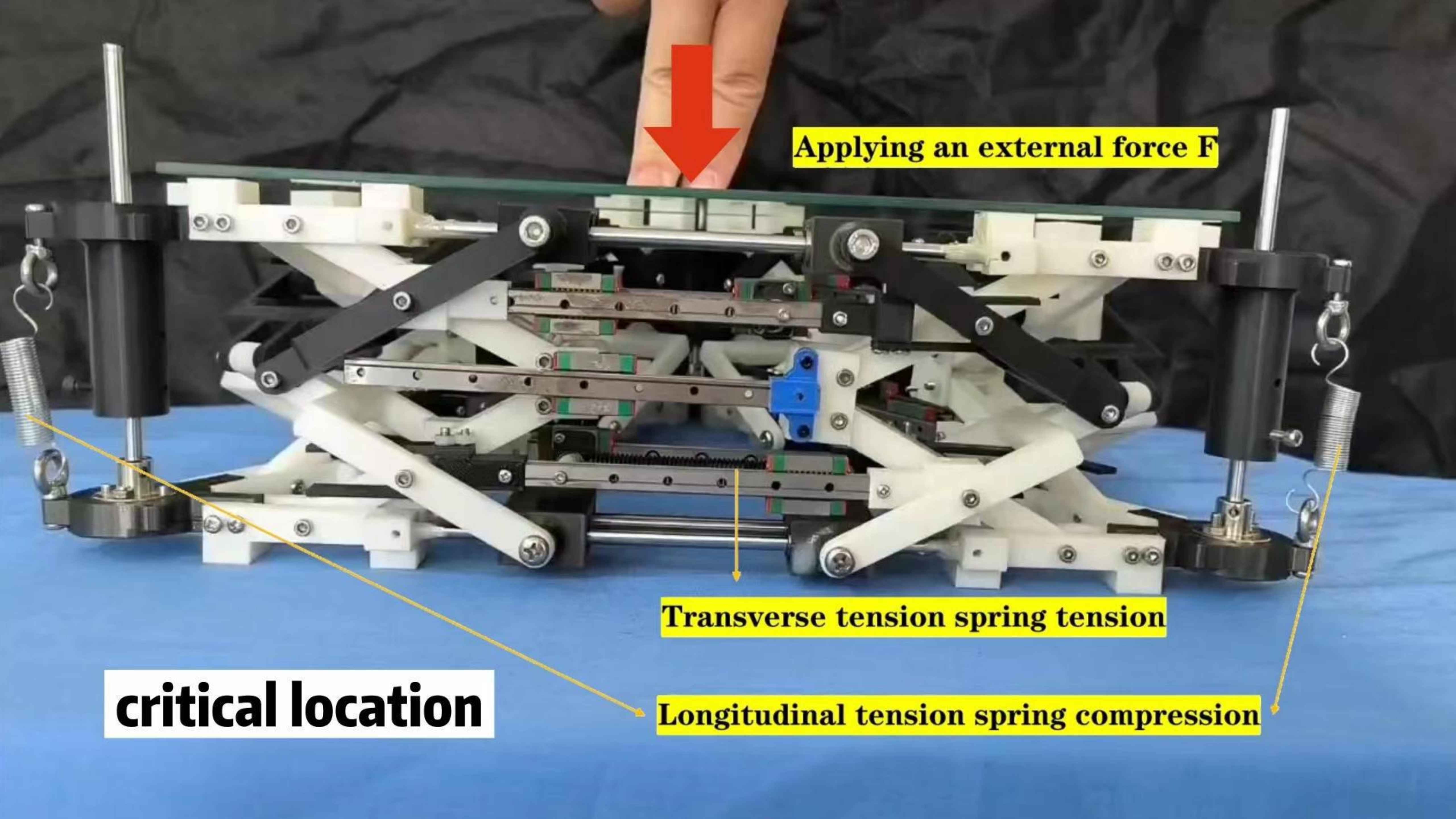

Applying an external force F
Transverse tension spring tension
critical location
Longitudinal tension spring compression

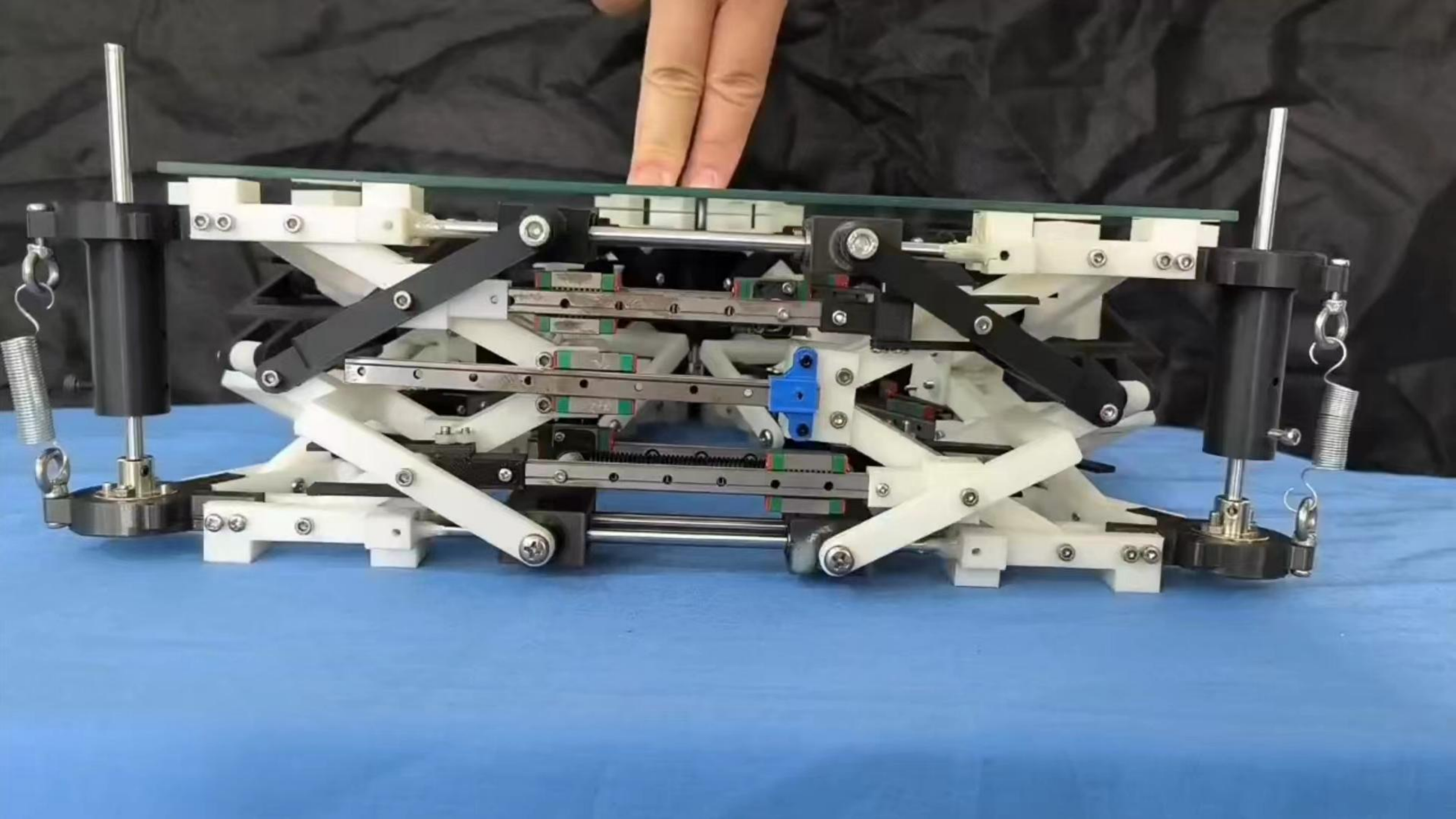

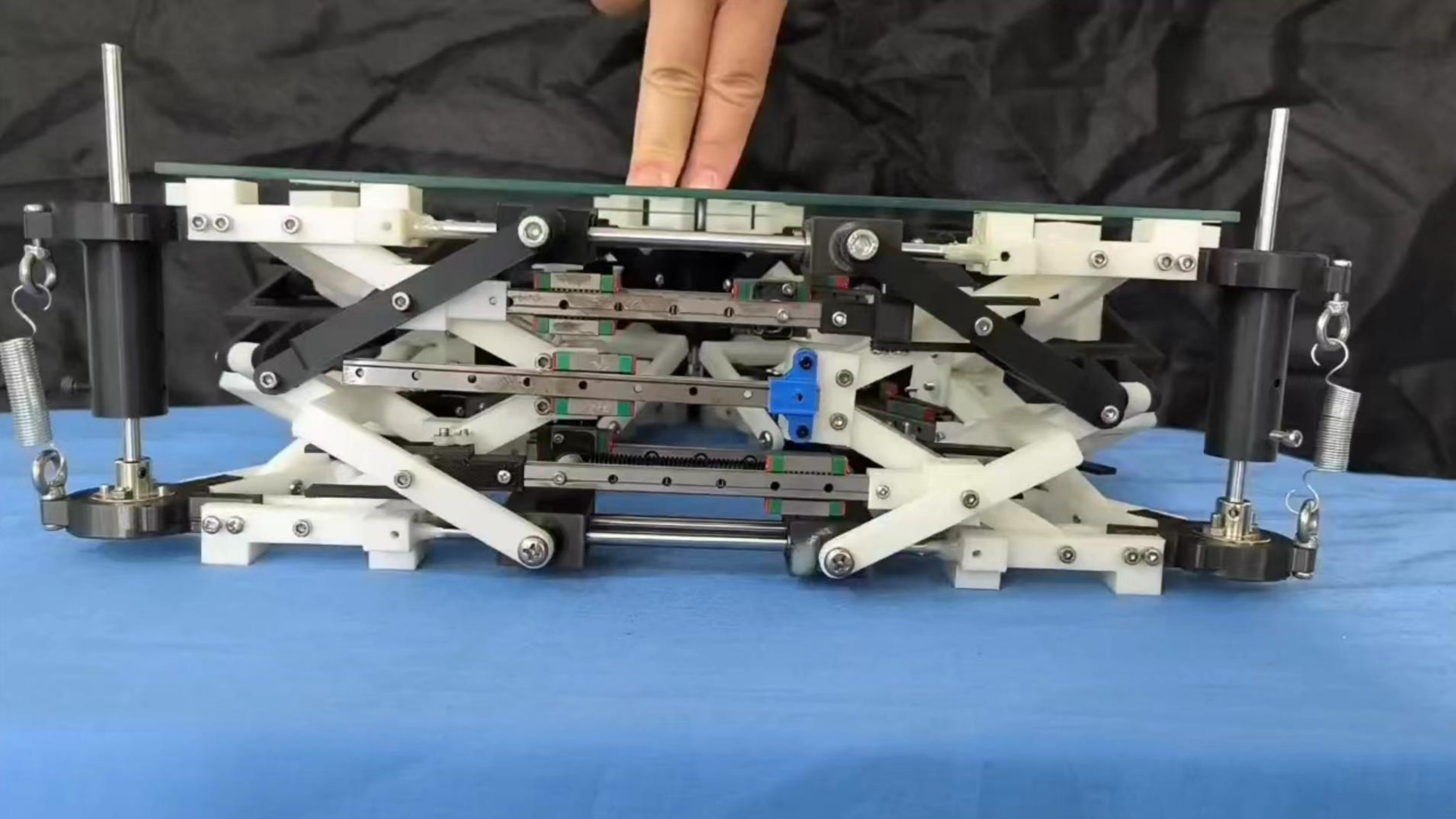

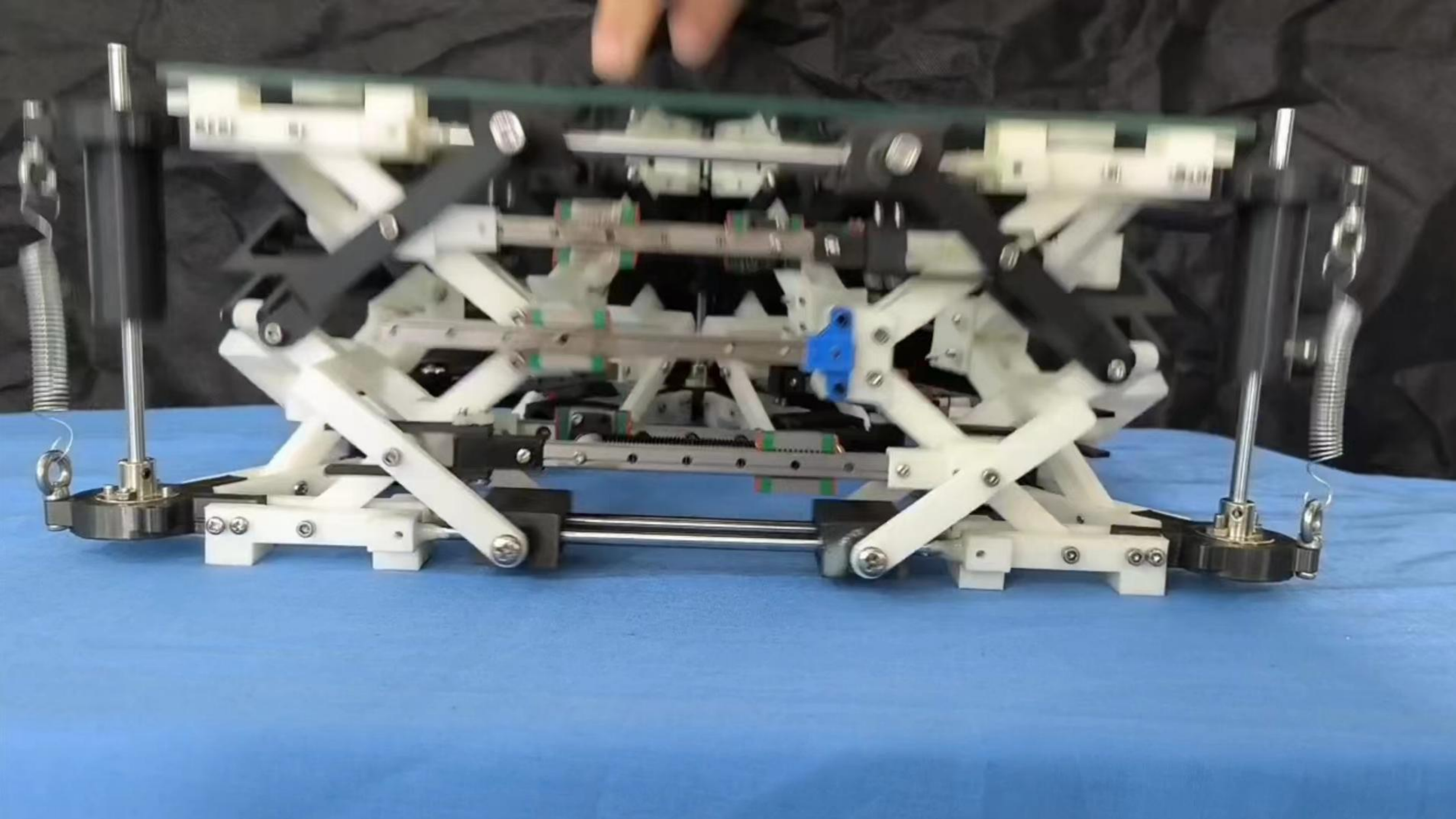

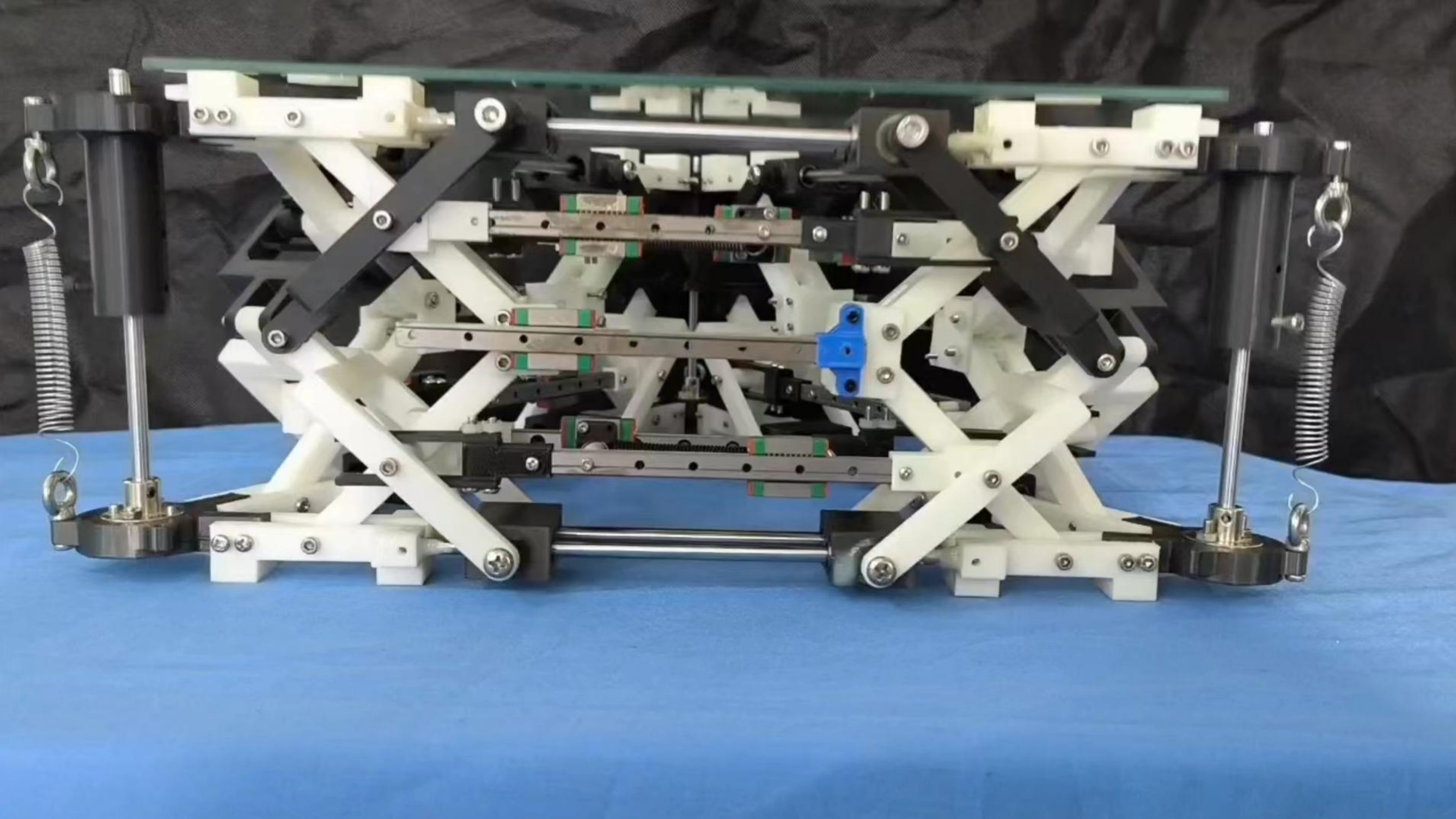

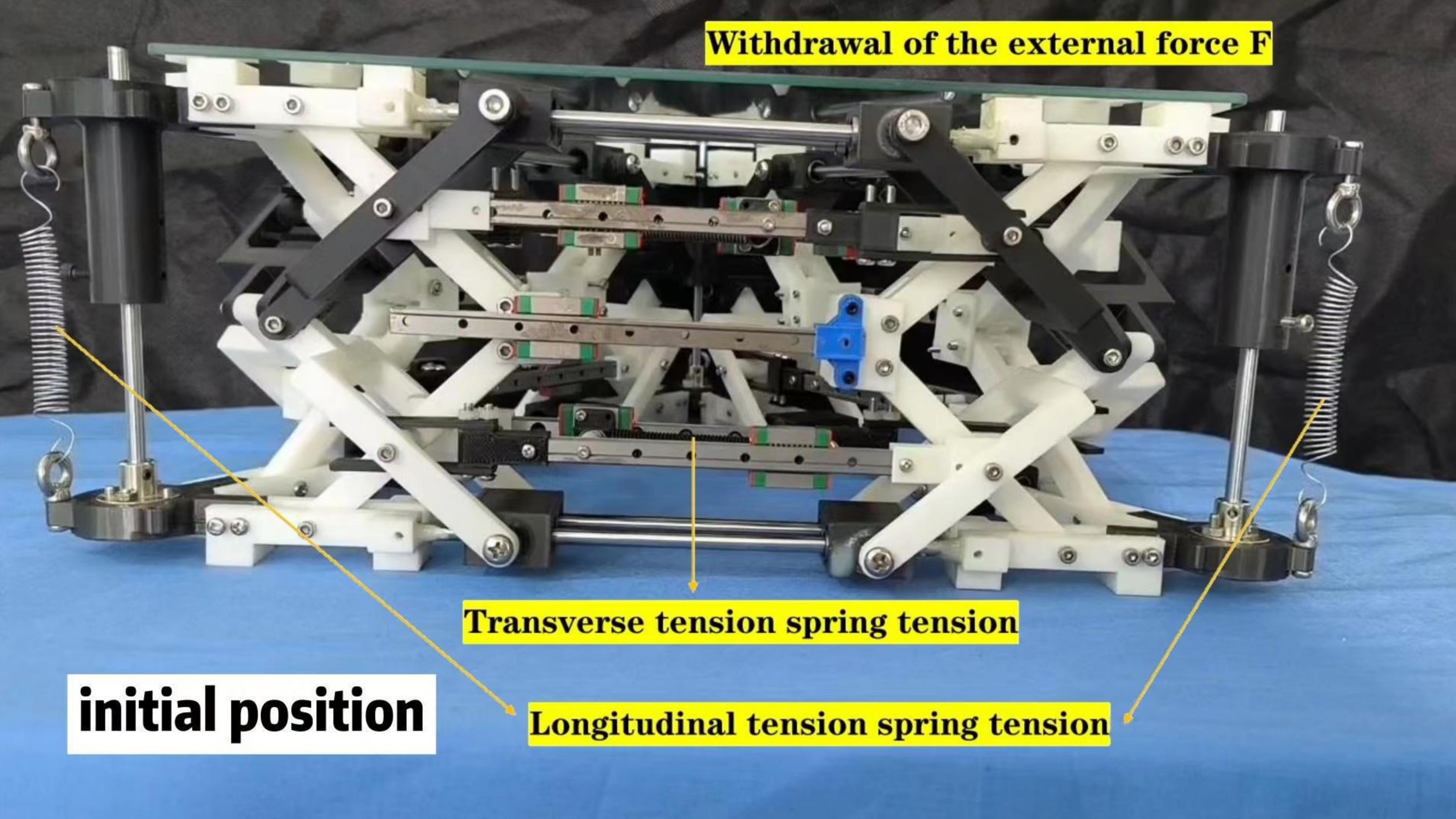
Withdrawal of the external force F
Transverse tension spring tension
initial position
Longitudinal tension spring tension

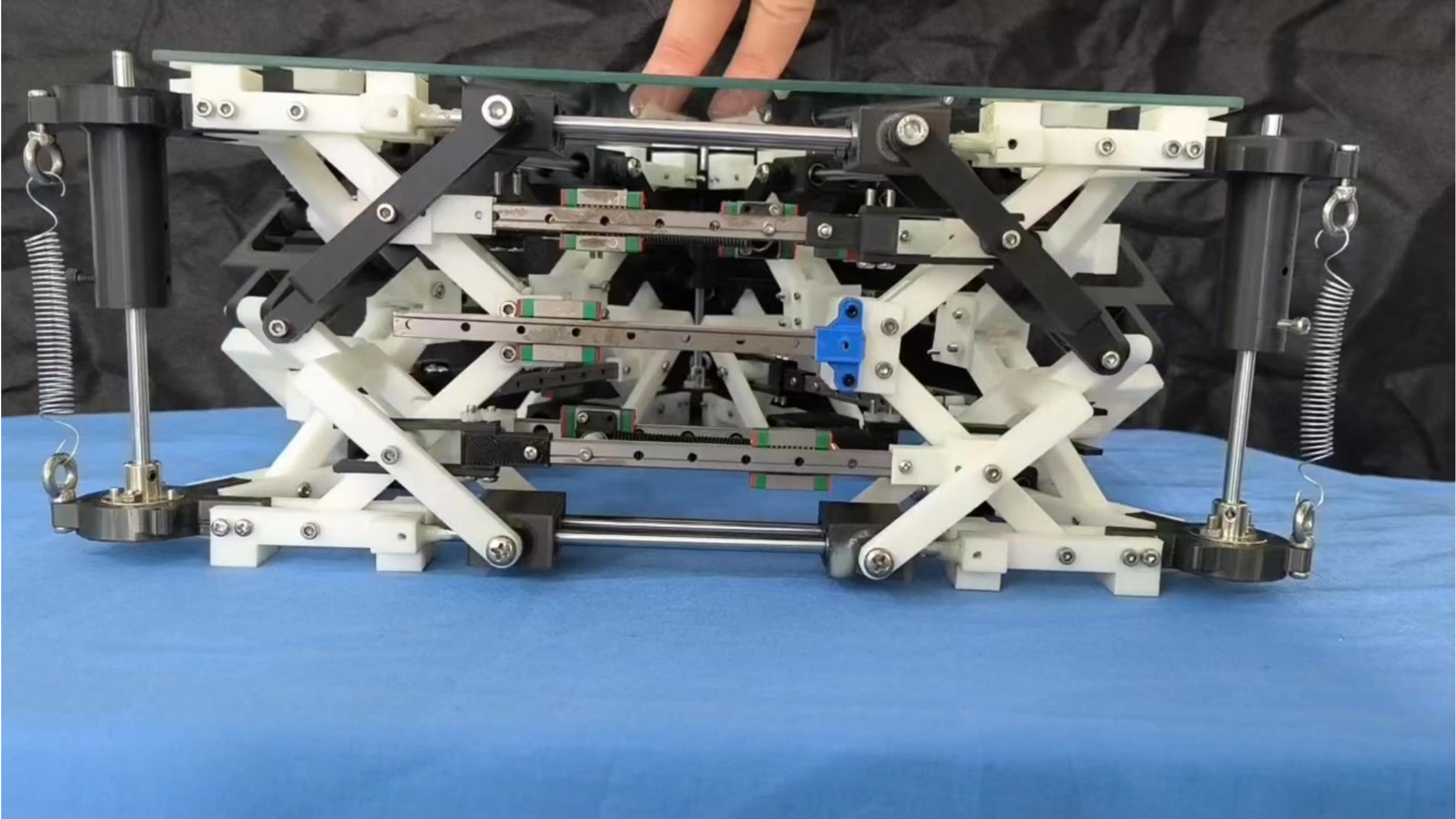

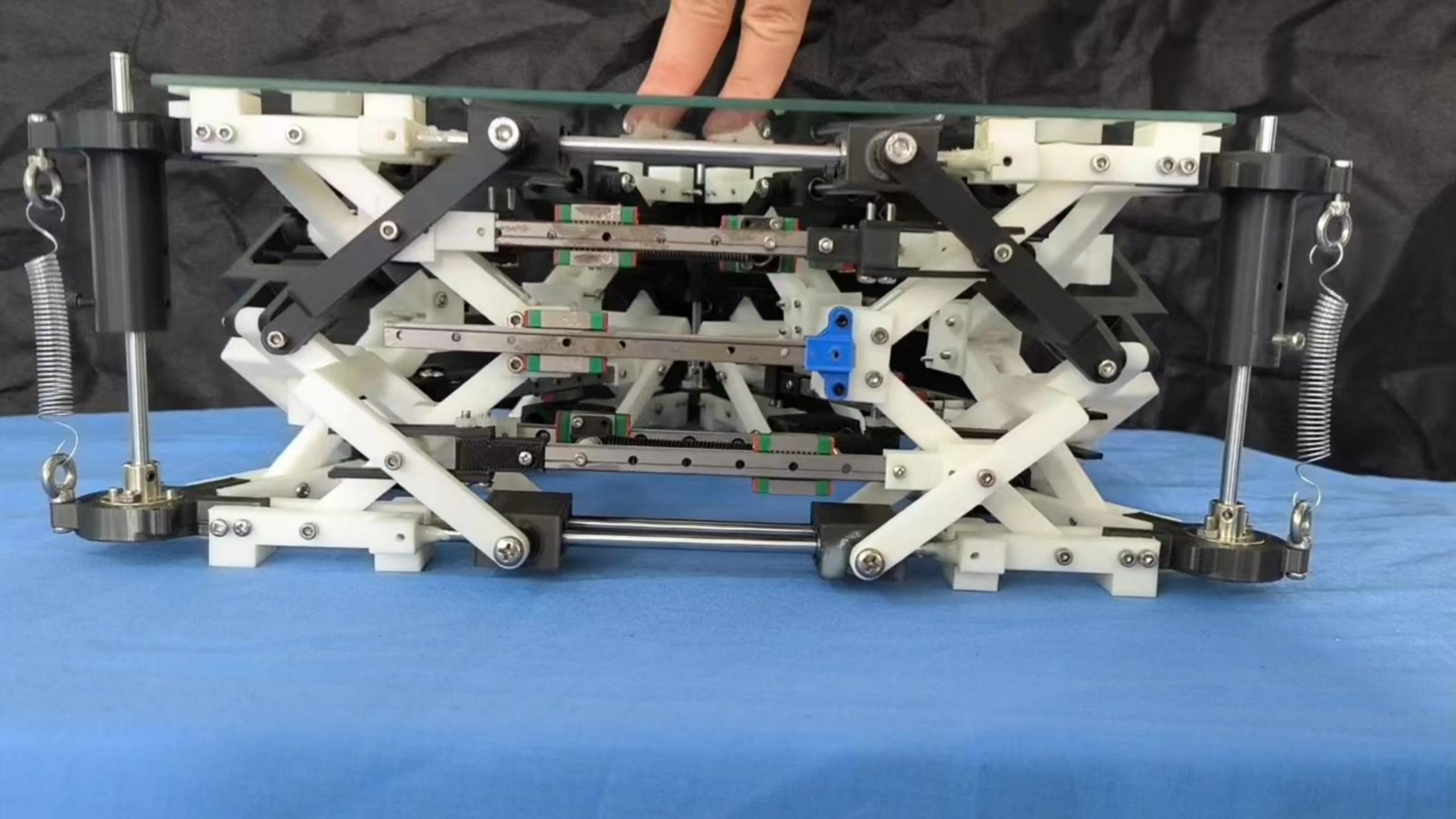

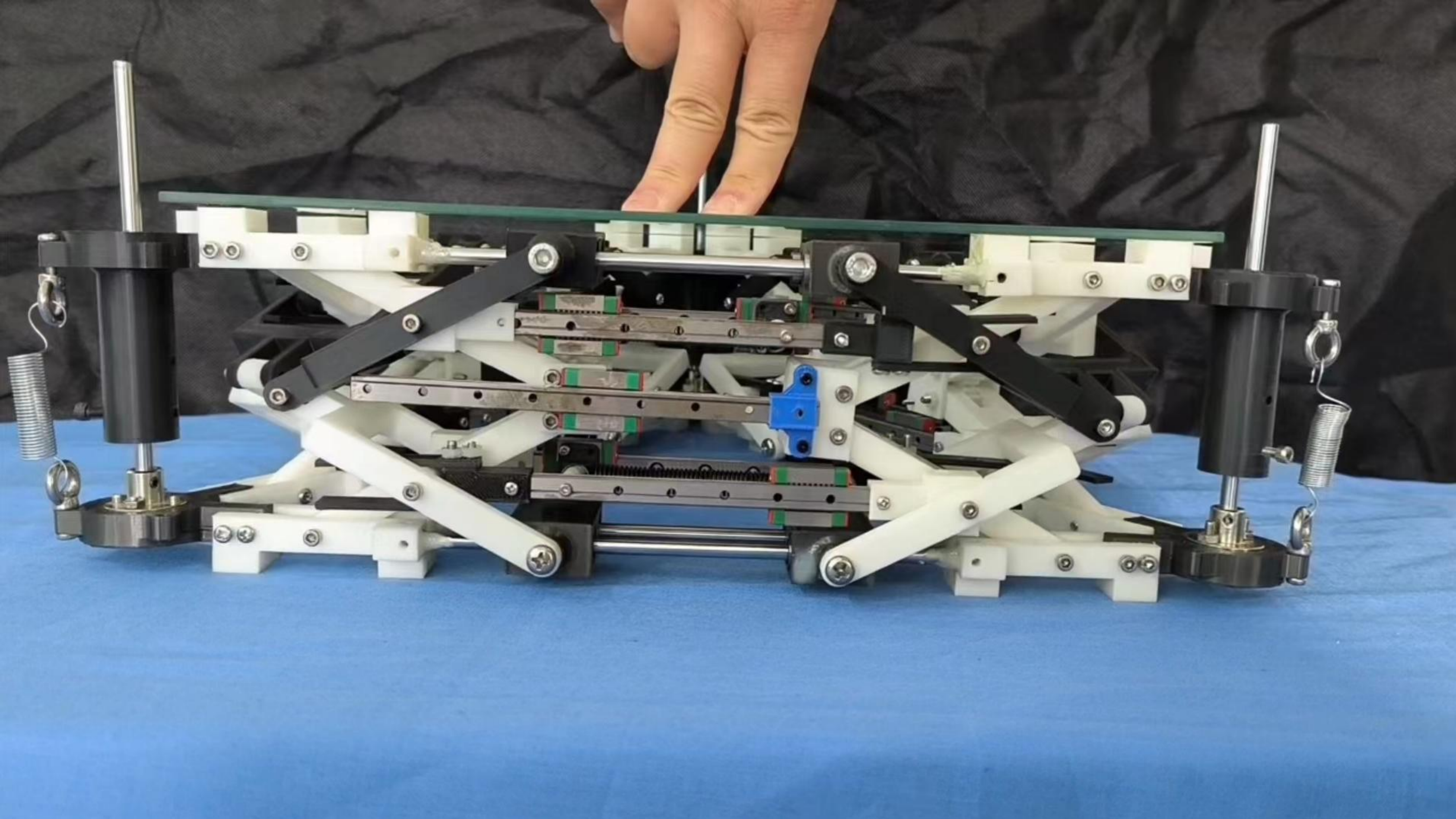

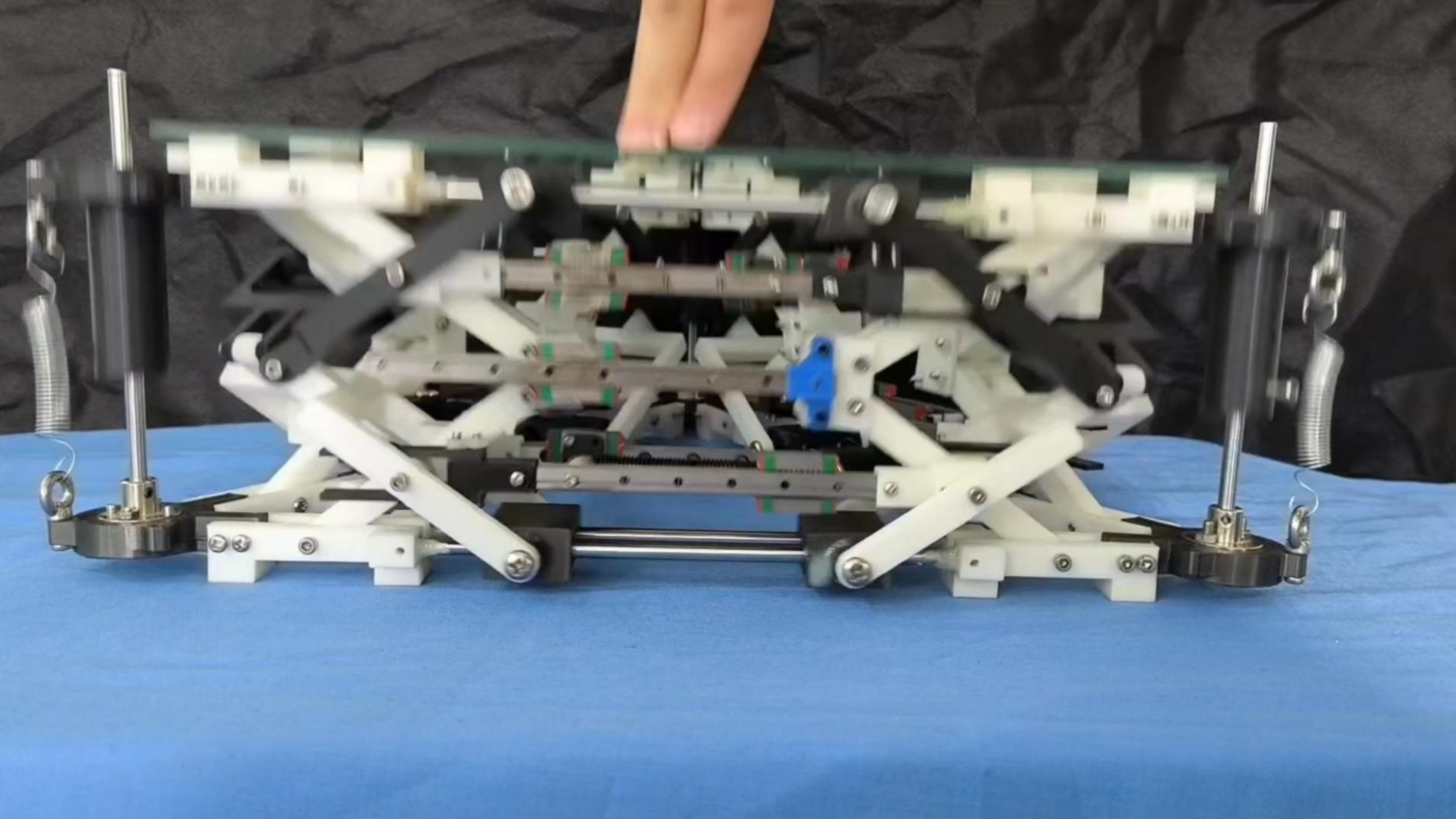

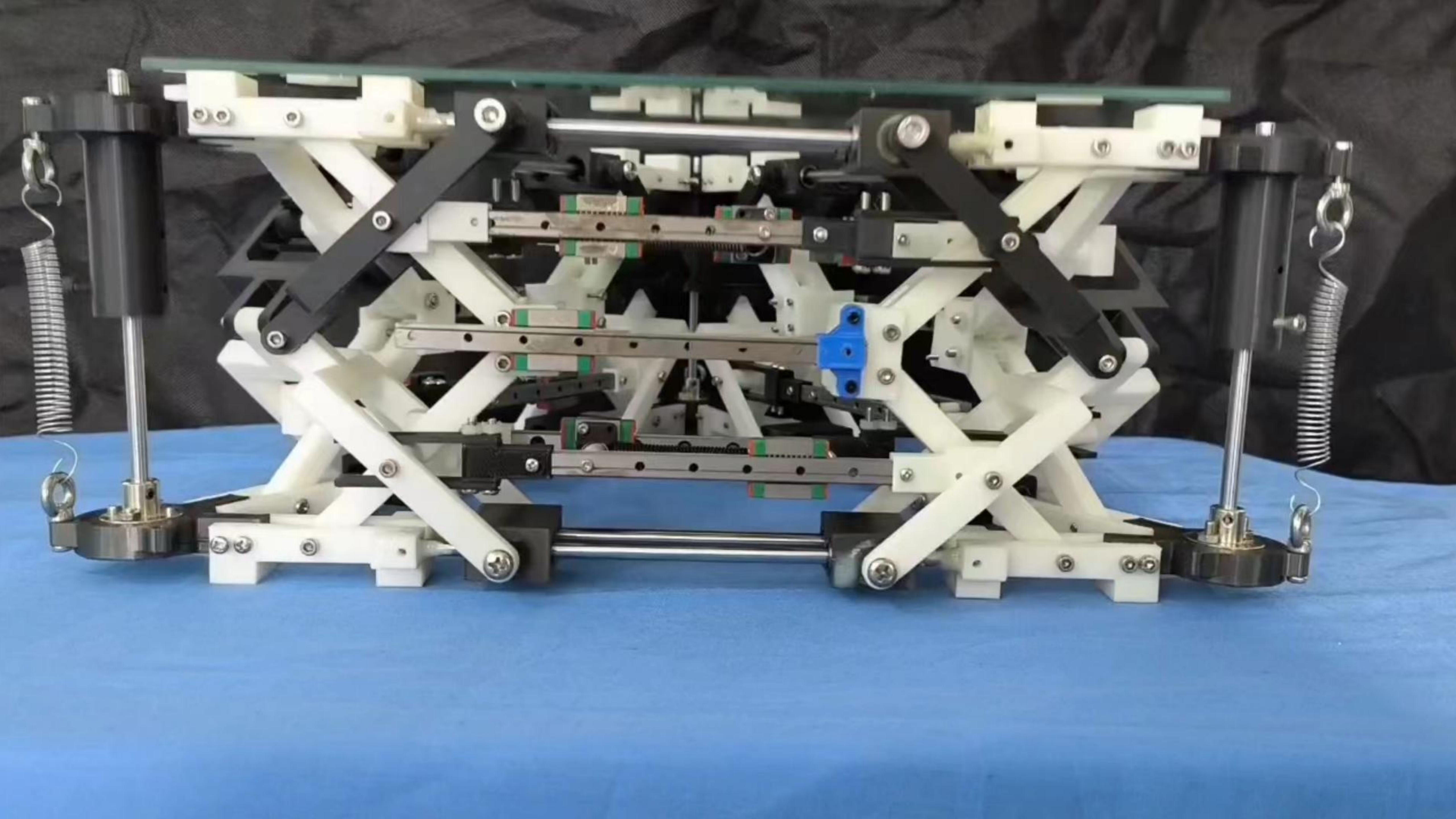

Tensioned extendable space single-layer extension wall includes: double-hole cylindrical slide, linear cylindrical optical axis, linear plain bearings, longitudinal tension springs, transverse tension springs, connecting rod mechanism, guide rails, sliding rods, flange couplings and so on.

In the initial position of the single-layer extension wall in tensile space, both the longitudinal and transverse extension springs are in the stretched state; when an external force is applied to the centre of the upper surface, under the action of the external force F, the upper platform moves downward, the longitudinal extension spring is compressed, and the transverse extension spring is continuously stretched; when the upper platform moves downward to the critical position, the external force F is kept unchanged, and the single-layer extension wall in tensile space continues to keep the state; when the external force F is withdrawn in a moment, the platform will be immediately restored to the initial position, and the transverse spring will be released, and the longitudinal spring will be stretched.
